# Elevated levels of Letm1 drives mitochondrial dysfunction and cardiomyocyte stress-mediated apoptosis in cultured cardiomyocytes

**DOI:** 10.1186/s12964-025-02378-7

**Published:** 2025-08-23

**Authors:** Anushka Deshpande, Leo Weirauch, Tapan Kumar Baral, Marco Steier, Ankush Borlepawar, Manju Kumari, Lucia S. Kilian, Karsten Richter, Elke Hammer, Derk Frank, Constanze Schmidt, Norbert Frey, Ashraf Y. Rangrez

**Affiliations:** 1 Department of Cardiology, Angiology and Pneumology, Internal Medicine III, University Hospital Heidelberg, Heidelberg, 69120 Germany; 2 DZHK (German Centre for Cardiovascular Research), partner site Heidelberg/Mannheim, Heidelberg, 69120 Germany; 3 Medical school, Hamburg, Germany; 4 Department of Cardiology and Internal Intensive Medicine, Internal Medicine III, University Hospital of Schleswig-Holstein, Kiel, Germany; 5 DZHK (German Centre for Cardiovascular Research), partner site Kiel/Hamburg/Lübeck, Kiel, 24105 Germany; 6 Core Facility Electron Microscopy, German Cancer Research Center (DKFZ), Heidelberg, 69120 Germany; 7 Interfaculty Institute of Genetics and Functional Genomics, University Medicine Greifswald, Greifswald, 17475 Germany; 8 DZHK (German Centre for Cardiovascular Research), Partnersite Greifswald, Greifswald, 17475 Germany

**Keywords:** Letm1, Mitochondrial metabolism, Cardiomyocytes, Hypertrophy, Arrhythmias

## Abstract

**Background:**

Cardiac ischemia, a predominant cause of heart failure, is marked by profound mitochondrial dysfunction, dysregulated ion homeostasis, and maladaptive cellular remodeling, all of which compromise cardiac performance. The mitochondrial inner membrane protein Leucine zipper-EF-hand containing Transmembrane Protein 1 (Letm1), implicated in Wolf-Hirschhorn Syndrome, is essential for mitochondrial function. Although genetic alterations in Letm1 are linked to cardiomyopathies, its specific contributions to cardiac pathophysiology, particularly in the context of ischemic heart disease, remain poorly defined. This study aims to elucidate the role of Letm1 in ischemic cardiac pathology and its mechanistic impact on cardiomyocyte function.

**Methods:**

Letm1 expression was assessed in human and murine models of heart failure due to ischemic cardiomyopathy (ICM) and cardiac hypertrophy. Letm1 was overexpressed in neonatal rat ventricular cardiomyocytes, adult mouse cardiomyocytes, and human induced pluripotent stem cell (iPSC)-derived cardiomyocytes to study mitochondrial function (Seahorse assays), structural and molecular remodeling (fluorescence microscopy, transmission electron microscopy (TEM), qPCR, immunoblotting), transcriptomic/proteomic profiles, calcium handling and electrophysiology (patch-clamp), autophagic flux (Bafilomycin A1, LC3-RFP-GFP), and cell survival.

**Results:**

Letm1 was markedly upregulated in ICM in both human and murine hearts, but unchanged in hypertrophic heart failure. Overexpression of Letm1 in cardiomyocytes resulted in profound mitochondrial dysfunction, including downregulation of oxidative phosphorylation (OXPHOS) genes, impaired membrane potential, reduced ATP output, increased proton leak, and elevated ROS levels. A metabolic shift toward glycolysis was observed, accompanied by reduced fatty acid oxidation. Electron microscopy revealed mitochondrial fragmentation, mitophagic vesicles, and sarcomeric disarray. Transcriptomic and proteomic analyses highlighted dysregulation of genes linked to mitochondrial organization, ion transport, and autophagy. Electrophysiologically, Letm1 reduced L-type Ca^2+^ current density and significantly shortened action potential duration, leading to impaired contractility. Letm1 overexpression activated upstream autophagy regulators (AMPK, ULK1) and enhanced LC3-II and p62 accumulation, but autophagic flux was impaired, as confirmed by LC3-RFP-GFP reporter and exacerbated by Bafilomycin A1 treatment. This dysregulated autophagy was coupled with mitochondrial stress, increased apoptosis (cleaved caspases), and reduced cardiomyocyte viability.

**Conclusion:**

This study indicates that Letm1 upregulation drives mitochondrial dysfunction, electrophysiology alterations, and activation of autophagy and apoptosis, culminating in cardiomyocyte injury in ischemic cardiomyopathy. By disrupting OXPHOS, calcium handling, and cell survival pathways, Letm1 contributes to ischemic remodeling and cardiac dysfunction. Targeting Letm1 presents a promising therapeutic strategy to alleviate ischemic damage and preserve cardiac function.

**Graphical abstract:**

This graphical abstract illustrates the multifaceted effects of elevated levels of Leucine zipper-EF-hand-containing transmembrane protein 1 (Letm1) on cardiomyocyte function. Increased Letm1 disrupts mitochondrial oxidative phosphorylation (OXPHOS), leading to energy supply deficits, mitochondrial dysregulation, and impaired ion channel activity. These alterations contribute to electrophysiological deficits and compromise cardiac action potential. Simultaneously, mitochondrial dysfunction accelerates autophagy and apoptosis, further diminishing cell survival. Together, these mechanisms drive contractile dysfunction in neonatal rat ventricular cardiomyocytes (NRVCMs), highlighting a critical role for Letm1 in cardiac pathophysiology.

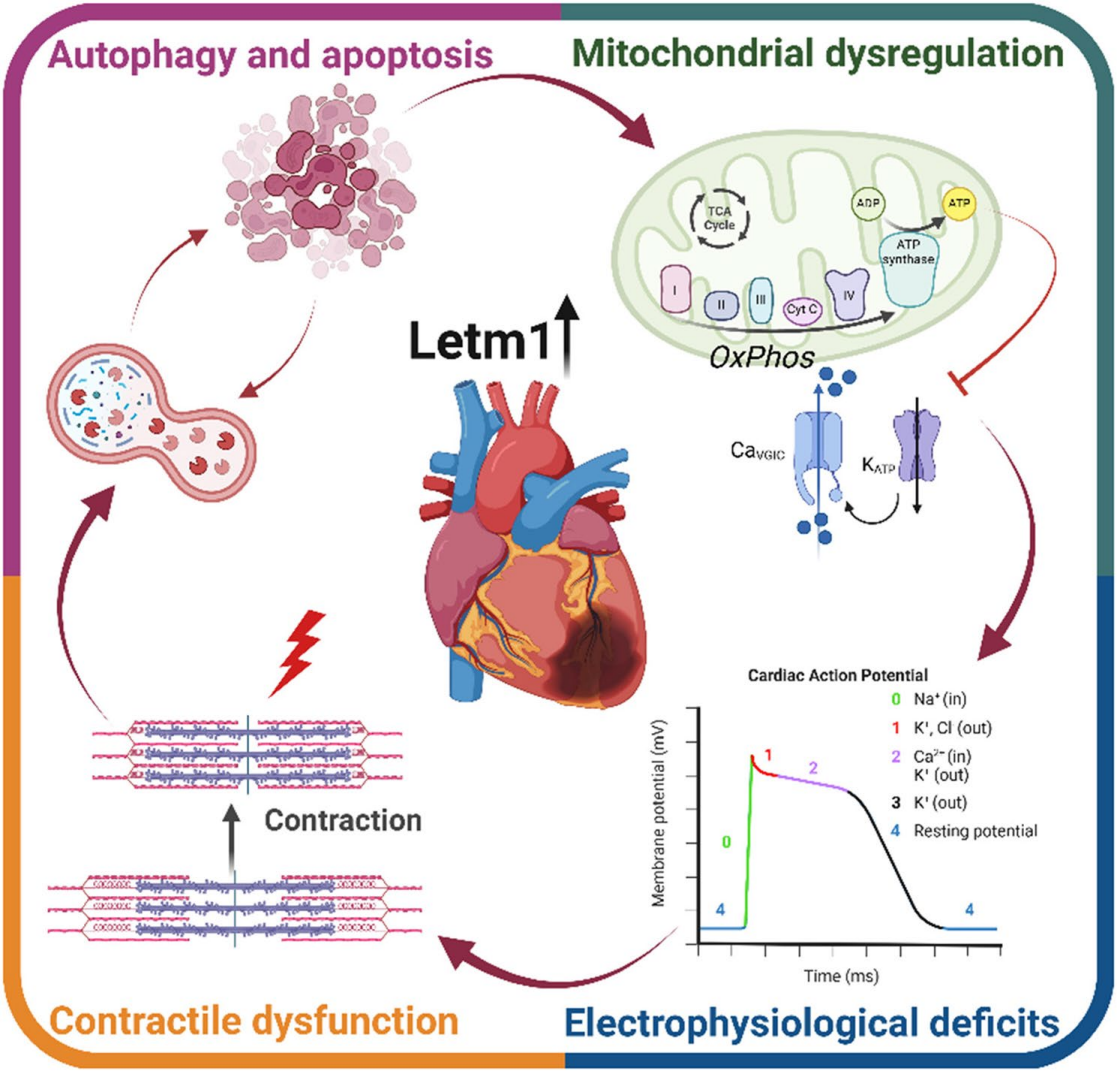

**Supplementary Information:**

The online version contains supplementary material available at 10.1186/s12964-025-02378-7.

## Background

Cardiovascular diseases (CVDs) remain the leading cause of mortality globally, with ischemic cardiomyopathy (ICM) representing the most prevalent and deadly form of cardiac dysfunction [1, 2]. ICM develops primarily from reduced coronary blood supply, resulting in myocardial ischemia, a condition characterized by insufficient oxygen and nutrient delivery to cardiac tissue. Prolonged ischemia, often followed by reperfusion, exacerbates cellular injury and initiates a cascade of pathological events that lead to cardiac remodeling, and eventually heart failure. The molecular underpinnings of ischemic injury are complex and multifactorial, encompassing oxidative stress, disrupted energy metabolism, ion imbalances, and maladaptive signaling pathways [3]. Despite advances in understanding the pathogenesis of ICM, the intricate mechanisms linking ischemic stress to cardiomyocyte dysfunction remain incompletely understood, posing challenges to developing effective therapeutic interventions. Mitochondrial dysfunction is central to the pathophysiology of ICM and plays a pivotal role in cardiomyocyte survival and energy homeostasis [4]. Mitochondria are the primary source of ATP in cardiomyocytes, supplying energy for contraction, relaxation, and ion transport [5]. Beyond their bioenergetic functions, mitochondria regulate cellular redox balance, apoptosis, and calcium signaling, which are critical to preserve cardiac function [5]. During ischemia, mitochondrial oxidative phosphorylation (OXPHOS) is impaired, leading to decreased ATP production and a shift toward glycolytic metabolism [4]. Concurrently, ischemia triggers an overproduction of reactive oxygen species (ROS), disruption of mitochondrial membrane potential (ΔΨm), and opening of the mitochondrial permeability transition pore, culminating in cell death [4, 6, 7]. These mitochondrial abnormalities are further amplified during reperfusion, as a burst of ROS and calcium overload exacerbate the mitochondrial injury and oxidative stress, promoting pathological remodeling of the myocardium [6, 8].

Mitochondrial dysfunction in ischemic cardiomyopathy extends beyond energy deficits, as it significantly affects ion homeostasis and electrophysiological stability. Calcium, a key regulator of excitation-contraction coupling, is meticulously modulated by mitochondrial buffering capacity and transport systems. Calcium cycling within cardiomyocytes is tightly orchestrated by calcium influx through L-type Ca^2+^ channels triggers ryanodine receptor 2 (RyR2)-mediated calcium release from the sarcoplasmic reticulum (SR), initiating contraction [9–12]. Relaxation is equally reliant on mitochondrial ATP to enable the reuptake of calcium by the sarcoplasmic/endoplasmic reticulum Ca^2+^-ATPase 2 (SERCA2) and its extrusion via the Na^+^/Ca^2+^ exchanger [13]. Mitochondria also serve as a buffer for excess cytosolic calcium, protecting cells from calcium overload that can precipitate oxidative damage and apoptosis [14]. However, ischemic stress disrupts this delicate balance, impairing calcium handling, altering ion channel expression, and causing contractile dysfunction [15–18]. These changes contribute to arrhythmia and progressive cardiac failure, highlighting the need to identify and target mitochondrial pathways that govern cardiomyocyte resilience under ischemic stress.

Emerging evidence suggests that mitochondrial dynamics and biogenesis are intricately regulated by specific proteins, expressions and function of which are found to be altered during ischemia. Among these, Leucine zipper-EF-hand containing Transmembrane Protein 1 (Letm1), a mitochondrial inner membrane protein, has garnered interest for its role in ion exchange and mitochondrial integrity. Initially identified in Wolf-Hirschhorn Syndrome [19]Letm1 is implicated in maintaining mitochondrial homeostasis through potassium, calcium, and proton exchange [19–21]. While its role has been extensively studied in other systems such as neuronal and cancer cells [22–28] the impact of Letm1 dysregulation on cardiomyocyte function and its contribution to cardiac diseases remain largely unexplored. Global deletion of Letm1 results in embryonic lethality, emphasizing its critical role in cellular and organismal viability [21]. Recent studies have identified rare bi-allelic missense and loss-of-function mutations in Letm1 associated with severe mitochondrial dysfunction and multi-organ failure, further highlighting its importance in human health [29]. Despite these findings, the specific role of Letm1 in cardiac physiology, particularly its gain-of-function effects, remains underexplored.

In this study, we uncovered a novel role of Letm1 in ischemic cardiomyopathy, demonstrating that it is upregulated in both human and murine models of ischemic heart disease. Due to the striking upregulation of Letm1 in ischemic heart and based on our knowledge of Letm1 loss-of-function being detrimental, here we explore for the first time the role of gain-of-function of Letm1 in cardiomyocyte pathophysiology. Surprisingly, as against expected protective effects, our results reveal that elevated Letm1 expression disrupts mitochondrial bioenergetics, ion homeostasis, and cellular survival mechanisms, culminating in cardiomyocyte dysfunction. By employing transcriptomic analyses, biochemical assays, and functional studies, we delineate the pathological consequences of Letm1 upregulation on oxidative phosphorylation, calcium handling, and stress response pathways. These findings shed light on the molecular mechanisms by which Letm1 likely contributes to ischemic remodeling and provides a basis for targeting this protein in therapeutic strategies aimed at mitigating ischemic injury and preserving cardiac function.

## Materials and methods

### Cloning and adenovirus generation

The expression construct for Letm1 was developed as described by Rangrez et al. [30]. The Letm1 overexpression construct was cloned from mouse heart cDNA using primers 5′-GCTGGCACCATGGCGTCCATCTTGCTCAGG-3′ and 3′-GCTGGGTCGCCCTAGTTCTTCACTTCTGCCGCC-5′. Sequential open reading frame (ORF) and adaptor PCRs were performed, and the purified PCR product was cloned into the pDONR221 vector (Gateway™ cloning system, Thermo Fisher Scientific, Germany) according to the manufacturer’s protocol. Adenoviruses encoding full-length mouse Letm1 cDNA were produced using the ViraPower™ Adenoviral Expression System (Thermo Fisher Scientific). After performing the LR clonase reaction to transfer the cDNA from pDONR221 into the pAd/CMV/V5-DEST vector, the construct was digested with PacI enzyme (10 U/µL; Thermo Fisher Scientific) and transfected into HEK293A cells for virus generation. Adenoviral titration was quantified by staining infected HEK293A cells with fluorescent anti-Hexon antibody. A β-galactosidase-expressing adenovirus (Ad-LacZ) served as the control. Both adenoviruses were used at a multiplicity of infection (MOI) of 50 infectious units (IFU) per cell for all experiments.

### Neonatal rat ventricular cardiomyocyte (NRVCM) isolation and culture

NRVCMs were isolated using established protocols as described before [31]. Briefly, left ventricles from 1-2-day-old Wistar rat pups (Charles River, Lyon, France) were excised, minced, and incubated in ADS buffer (120 mmol/L NaCl, 20 mmol/L HEPES, 8 mmol/L NaH_2_PO_4_, 6 mmol/L glucose, 5 mmol/L KCl, 0.8 mmol/L MgSO_4_, pH 7.4). Dissociation into single cells was achieved through five to six rounds of enzymatic digestion at 37 °C with pancreatin (0.6 mg/mL; Sigma) and collagenase type II (0.5 mg/mL; Worthington, Columbus, OH, USA). The cell suspension was filtered, and newborn calf serum was added to halt enzymatic activity. Cardiomyocytes were enriched through Percoll gradient centrifugation (GE Healthcare, Chicago, IL, USA) to minimize contamination with fibroblasts. Cells were cultured in DMEM containing 10% fetal calf serum (FCS), 2 mM penicillin/streptomycin, and L-glutamine (PAA Laboratories, Austria) and transduced with adenoviruses 24 h post-isolation and incubated in a humidified atmosphere at 37 °C and 5% CO_2_ in a cell incubator. Transduced NRVCMs were incubated in serum-free DMEM with penicillin/streptomycin and L-glutamine, and samples were collected 72 h post-transduction. For hypoxia experiments, cells were subjected to a 1% oxygen environment for the final 12 h of incubation (from hour 61 to hour 72) prior to harvesting them for downstream analyses. For Bafilomycin A1 (SantaCruz Biotechnology, #SC201550) treatment, the cells were treated at a final concentration of 50nM for 12 h before termination. LC3-GFP-RFP construct was transduced in combination with LacZ and Letm1 following 72 h, cells were fixed and counterstained with DAPI before imaging.

### Adult mouse cardiomyocyte (AMCM) isolation and culture

Adult mouse cardiomyocytes were isolated from C57BL/6 N wildtype mice as described in [32]. Briefly, animals were anesthetized, and hearts were perfused via the right ventricle using a 27-gauge needle at a flow rate of 1 mL/min with calcium-free EDTA buffer. Following perfusion, the ascending aorta was clamped, and the heart was transferred to a 60 mm Petri dish. The left ventricle was then perfused from the apex with EDTA buffer at the same flow rate, followed by perfusion buffer as outlined in the referenced protocol. Enzymatic digestion was carried out using 3–5 sequential digestions with collagenase II (Worthington Biochemical), continued until the myocardium became visibly pale and lost rigidity. The right ventricle was removed, and the remaining tissue was minced into ~ 1 mm^3^ fragments to facilitate dissociation. Cells were liberated by gentle trituration, and digestion was halted by adding STOP buffer (5% FCS in perfusion buffer). Cardiomyocytes were allowed to settle in a humidified incubator, and calcium was gradually reintroduced using M199 buffer base and perfusion buffer, according to the published protocol. Cells were then plated onto laminin-coated dishes (5 µg/mL) in plating medium and maintained in culture medium for downstream experiments. For gene delivery, adenoviral infection was performed at 5000 IFU per cell for 36 h to achieve transgene expression.

### Transmission electron microscopy

Primary cardiomyocytes collected from wildtype mice (adult) were cultured on laminin-coated glass coverslips, virally transduced with either Letm1 or LacZ (control), and were fixed for TEM-investigation 36 h after transfection. After primary fixation in buffered aldehyde (2% formaldehyde, 2% glutaraldehyde, 1mM MgCl_2_ in 100mM Ca-Cacodylate, pH 7.2), samples were postfixed in buffered osmium tetroxide (1% in 40mM Ca-Cacodylate) followed by en-block staining in uranylacetate (1% in 75% Ethanol (70%), dehydration in graded steps of ethanol and embedding Epoxide (Glycidether, NMA, DDSA: Serva, Heidelberg, Germany). Ultrathin sections at nominal thickness 60 nm and contrast-stained with lead-citrate and uranylacetate were observed in a Zeiss EM 910 at 80 kV (Carl Zeiss, Oberkochen, Germany) and micrographs taken using a digital CCD camera (TRS, Moorenweis, Germany).

### Differentiation of human induced pluripotent stem cells into cardiomycoytes

Human iPSC-derived cardiomyocytes (hiPSC-CMs) were obtained as cryopreserved plates from the Stem Cell Core Facility (Dr. Timon Seeger). For differentiation, Matrigel-coated 6-well plates were prepared 2 h before seeding. Cells were thawed on ice, gently centrifuged (300 × g, 3 min), and resuspended in hE8 medium with ROCK inhibitor (Y-27632) before plating. Cultures were maintained at 37 °C with 5% CO_2_. Cardiomyocyte differentiation was carried out using a chemically defined protocol [33]. On day 0, cells were treated with CDM3 medium containing CHIR99021. On day 2, medium was replaced with CDM3 alone. From day 3–4, CDM3 was supplemented with IWP2, followed again by CDM3 alone from day 5–6. From day 7 onward, cells were cultured in B27-supplemented medium (with insulin), followed by glucose-depleted CDM3 for metabolic selection (day 10–12). On day 15, cells were passaged and maintained in B27 + ins medium. All experiments were conducted using day-40 hiPSC-CMs.

### Proteomics analysis

Four bioreplicates were analyzed across four experimental groups (LacZ, Letm1). Samples were lysed by five freeze-thaw cycles at 30 °C and 1400 rpm in 8 M urea/2 M thiourea buffer. Lysates were centrifuged at 16,000 × g for 1 h at room temperature, and protein concentrations were determined using the Bradford assay with BSA as the standard. DNA was fragmented via sonication. Four micrograms of protein per sample were processed using a modified SP3 bead-based protocol [34]. Proteins were reduced, alkylated, and digested with LysC/Trypsin (1:25) overnight at 37 °C. Digestion was quenched with 0.1% TFA, and peptides (5 µL of 22 µL total) were loaded onto Evotips per manufacturer instructions. Peptides were separated on an Evosep One system using a 15 cm × 150 μm, 1.5 μm Performance column and a 30 samples/day gradient with buffer A (0.1% formic acid in water) and buffer B (0.1% formic acid in acetonitrile). Data-independent acquisition (DIA) was performed on a TimsTOF HT mass spectrometer. Data was analyzed in Spectronaut v19.9 using directDIA (library-free) with Prosit spectral predictions. Protein inference was based on “Protein Group ID” and peptides on “Stripped Sequence.” Only proteins with ≥ 2 peptides and precursors present in > 50% of samples per condition were quantified (Q-value < 0.01). Ion-level data were median-normalized (MS2 total peak area), excluding Met-oxidized peptides. Peptide intensities were calculated by summing ion intensities, excluding shared peptides. Protein intensities were determined using MaxLFQ algorithm. Protein log2 fold changes were derived from median peptide intensities. Figures were made using ggplot2.

### Co-localization of Letm1 with mitotracker

Letm1 localization with mitochondria was assessed in AMCMs using confocal microscopy (Zeiss LSM800, ZEN-blue software). After 72 h of adenoviral transduction, cells on collagen-coated coverslips were stained with 200 nM MitoTracker Red (Thermo Fisher Scientific) for 15 min, washed with PBS, and fixed with 4% paraformaldehyde (PFA) for 5 min. After permeabilization (0.1% Triton X-100, 2.5% BSA in PBS), cells were incubated with rabbit polyclonal anti-Letm1 antibody (1:200 dilution, SantaCruz) for 1 h, followed by Alexa Fluor-488-conjugated secondary antibody (1:200 dilution, Thermo Fisher Scientific) and DAPI (1:1000 dilution). Fluorescence images were acquired at room temperature using a Plan-Apochromat 40x/1.4 oil objective. Detector gain and laser power were optimized for each fluorophore, ensuring precise co-localization analysis.

### Immunofluorescence staining for cell size measurement and LC3 fluorescence measurement

Cell size analysis was performed by immunofluorescence microscopy as previously described [30] using a monoclonal mouse anti-α-actinin antibody (1:200, Sigma). Cells were incubated with Alexa Fluor-488 secondary antibody (1:200, Thermo Fisher Scientific) and DAPI, mounted with FluorSave™ reagent (Merck Millipore), and imaged with a Keyence BZ-9000 fluorescence microscope (10x objective, NA: 0.45). Images (10 per coverslip) were analyzed with Keyence BZ-II Analyzer software. The HybridCellCount module was employed to define cardiomyocyte boundaries based on α-actinin fluorescence, filter for single-nucleus cells (200–2500 μm² area with a nucleus-to-surface ratio < 30%), and exclude apoptotic cells. All quantifications were performed using randomized fields from matched-density cultures, and the decrease in cell area was statistically significant across biological replicates. For LC3 GFP-RFP, cells were imaged using MICA miscroscope (Leica) post DAPI counterstain and the data was analysed using ImageJ software.

### TMRE measurement

To assess mitochondrial membrane potential, NRVCMs were incubated with 200 nM tetramethylrhodamine ethyl ester (TMRE) in live-cell conditions for 30 min at 37 °C. Following incubation, the cells were washed twice with pre-warmed media to remove excess dye and then imaged using a Zeiss LSM800 confocal microscope equipped with ZEN Blue software. Imaging was performed at 37 °C using a Plan-Apochromat 40x/1.4 oil immersion objective. Detector-gain and laser power were initially optimized for the control condition, and subsequent experimental conditions were imaged under identical software and hardware settings to ensure consistency. TMRE fluorescence intensity was quantified using ImageJ software, and values were normalized as appropriate for downstream analyses.

### MitoSOX staining

MitoSOX Red was utilized to detect mitochondrial reactive oxygen species (ROS) in NRVCMs. Cells were incubated with 50 nM MitoSOX Red, diluted in HBSS, for 30 min at 37 °C. After incubation, cells were washed thoroughly to remove unbound dye, fixed with 4% PFA for 15 min, and subsequently stained with DAPI to counterstain nuclei. Imaging was performed using the Zeiss LSM800 confocal microscope and analyzed with ZEN Blue software. Detector settings were kept consistent across experimental conditions to ensure reliable comparisons. Quantitative fluorescence analysis was conducted using ImageJ.

### MTT assay for cell viability

NRVCMs cultured in 24-well plates for viability analysis were infected with Letm1 adenoviruses, using LacZ as the control, and incubated for 72 h under conditions specified above. An MTT labeling reagent (In Situ Cell Proliferation Kit, MTT I, Roche Applied Science, Penzberg, Germany) was added at 10% of the total DMEM volume per well. The plates were incubated for 4 h at 37 °C. Following the initial incubation, an MTT solubilization solution (Cell Proliferation Kit MTT, Roche Applied Science) was added at 10 times the volume of the MTT labeling reagent. The plates were further incubated overnight under the same conditions to allow complete solubilization of purple formazan crystals. Spectrophotometric absorbance was measured using a 96-microplate reader on an Infinite M200 PRO System (Tecan, Life Science). Cell viability was calculated as a percentage relative to the negative control, and statistical comparisons between groups were performed using the Student’s t-test. All experiments were conducted in eight to twelve replicates and repeated in three independent experiments.

### RNA isolation and quantitative RT-PCR

Total RNA from NRVCMs, AMCMs, iPSCs and tissue samples was extracted with TRIzol™ reagent (Thermo Fisher Scientific) and reverse-transcribed using the LunaScript™ RT SuperMix Kit (New England Biolabs). qRT-PCR was performed using SyBr Green Mastermix (Applied Biosystems) on a Roche LightCycler 480. Rpl32 as a housekeeping gene served as internal control, and relative quantification was determined using the ∆∆Ct method. All reactions were conducted in hexaplicates and repeated thrice.

### DNA isolation and quantitative RT-PCR

Total DNA from NRVCMs, AMCMs and tissue samples was extracted using Phenol-Choloroform-Iso-amyl alcohol (PCI) method and post quantification, qRT-PCR was performed using SyBr Green Mastermix (Applied Biosystems) on a Roche LightCycler 480. 18 s rRNA, β-actin or HK2 were used as nuclear genes to normalize against Cytb or ND1 mitochondrial genes. relative quantification was determined using the ∆∆Ct method. All reactions were conducted in hexaplicates.

### Differential gene expression analysis

RNA-seq was performed at Novogene Co. Reads were filtered, normalized, and analyzed for differential expression using DESeq2 [35]. Genes with adjusted *p*-values (padj) ≤ 0.01 and log_2_ fold changes ≥ 0.5 or ≤−0.5 were considered differentially expressed. Heatmaps and volcano plots were generated with ComplexHeatmap [36] and EnhancedVolcano [37] R packages, respectively. Gene ontology (GO) and KEGG pathway enrichment analyses were conducted using clusterProfiler [38]with FDR thresholds of ≤ 0.05 for GO terms and ≤ 0.1 for KEGG pathways.

### Protein preparation and immunoblotting

NRVCMs were lysed using RIPA buffer (50 mM Tris, 150 mM NaCl, 1% Nonidet P-40, 0.5% sodium deoxycholate, 0.2% SDS) supplemented with phosphatase inhibitors II and III and a protease inhibitor mix (Roche Applied Science). Cell lysis was achieved through 2–3 freeze-thaw cycles. Mouse or human heart tissue proteins were extracted using a Precellys homogenizer equipped with coarse and fine ceramic beads (Peqlab, Germany). Cell debris was removed by centrifugation, and protein concentrations were determined photometrically using the DC assay (Bio-Rad, Feldkirchen, Germany) against BSA standards. Proteins were separated by 10% SDS-PAGE, transferred to nitrocellulose membranes, and immunoblotted with primary antibodies, including: Letm1 (1:1000, Santa Cruz Biotechnology SC271235), OXPHOS antibody cocktail rodent (1:5000, Abcam 110413), Caspase 3 (1:1000, Cell Signaling Technology, 9662), Caspase 7 (1:1000, Cell Signaling Technology 9492), α-actinin (1:2000, Sigma-Aldrich, A7732), Cpt1a (1:1000, Proteintech, 66039-1-Ig), ULK1 (1:1000, CST, 8054), pULK1 (1:1000, CST, 5869), AMPK (1:1000, CST, 2534), DRP1 (1:1000, CST, 5391), pDRP1 ser 616 (1:1000, 4494), LAMP2 (1:1000, GTX635790), BNIP3L (1:1000, GTX64447), p62 (1:1000, CST, 5114), LC3 (1:1000, CST, 2775). After overnight incubation with primary antibodies, membranes were incubated with HRP-conjugated secondary antibodies (1:10,000; Santa Cruz Biotechnology) or Alexa Fluor 546 fluorescent antibodies. Protein bands were visualized using a chemiluminescence detection kit (GE Healthcare) and imaged on a ChemiDoc system (Bio-Rad). Quantitative densitometry was performed using ImageJ software (version 1.46, NIH), and results were normalized to controls using GraphPad Prism. Experiments were performed in triplicates and repeated three times.

### Human heart samples

Left ventricular myocardial samples were collected from explanted hearts of patients with end-stage heart failure (New York Heart Association Class IV) undergoing heart transplants. Samples included non-failing (NF), ischemic cardiomyopathy (ICM), and hypertrophic cardiomyopathy (HCM) patients. Ethical guidelines were followed as approved by the Medical School’s Ethics Committee at the University of Göttingen, Germany. Samples were placed in pre-cooled cardioplegic solution (composition: NaCl 110 mM, KCl 16 mM, MgCl_2_ 16 mM, NaHCO_3_ 16 mM, CaCl_2_ 1.2 mM, glucose 11 mM) immediately after surgery to preserve tissue viability. For immunoblotting, tissues were flash-frozen in liquid nitrogen and stored at −80 °C.

### ORAB mice heart samples

Twelve-weeks-old mice received an intraperitoneal injection of buprenorphine (0.05–0.1 mg/kg) 30 min before surgery. During cardiac surgery, animals were intubated via a rigid orotracheal tube connected to a ventilator. A 1–1.5 cm incision was made on the left thorax, exposing the ascending aorta. A nitrile rubber O-ring, modified with a 6 − 0 non-absorbable suture, was passed under the aorta, tightened, and secured with a knot. The chest and skin were sutured. Sham surgeries involved the same procedure without O-ring placement. Post-surgery, mice were housed individually and if necessary, kept on heating pads to maintain temperature regulation to prevent hypothermia, and received tramadol (2.5 µg/mL) in drinking water for 2–3 days. Echocardiography was carried out 4 weeks post-surgery, followed by organ harvesting for downstream applications.

### LAD mice heart samples

Ligation of the left anterior descending coronary artery (LAD) was performed under general anesthesia with 3% isoflurane. Buprenorphine (0.1 mg/kg) was administered pre-operatively, and 2% lidocaine (0.2–0.3 mL) was applied locally at the incision site. A craniocaudal incision was made on the left sternum, and retractors were used to expose the thoracic cavity. The LAD was ligated using an 8 − 0 Prolene suture. The air was evacuated from the thoracic cavity with a suction drain, and the chest and skin were sutured. Post-surgery care included regulated warming and tramadol (2.5 µg/mL) in drinking water for 2–3 days. Echocardiography confirmed successful ligation, and organs were harvested post 4-weeks of surgery.

### Glucose uptake assay

Glucose uptake in NRVCMs was assessed following established protocols [39, 40]. Briefly, NRVCMs were treated under experimental conditions for 72 h, after which they were incubated in serum-free DMEM (Gibco, Cat. No. 11-965-118) for 15 min to synchronize metabolic activity. Subsequently, cells were treated with 0.5 µCi/mL of 2-Deoxy-D-glucose, [1,2-^3^H(N)] (Hartmann Analytic, Cat. No. MT911) for 15 min to measure glucose uptake. The uptake reaction was terminated by adding ice-cold medium containing 10 µM cytochalasin B (Sigma) to inhibit further glucose transport. To quantify uptake, cells were lysed with 0.1 N NaOH for 20 min at room temperature. The lysates were then analyzed for radioactivity using a scintillation counter. Protein concentrations were measured using the BCA Protein Assay Kit (Pierce), and glucose uptake rates were normalized to the protein content in each sample.

### Patch-clamp electrophysiology

Borosilicate glass pipettes (1B120F-4, World Precision Instruments, Berlin, Germany) with resistances of 6–13 MΩ were used. Recordings were obtained with an Axopatch 200B amplifier (Axon Instruments, USA) interfaced with an Axon Digidata 1550B digitizer, and data were analyzed using pCLAMP 11 software. All experiments were conducted at room temperature (22–25 °C).

#### Action potential (AP) measurements

APs were recorded from murine atrial cardiomyocytes (CMs) in current-clamp mode. Cells were stimulated with 3 ms current pulses (250–800 pA) at 0.5 Hz, applying an average holding current density of −0.97 ± 0.03 pA/pF. The intracellular solution contained (in mmol/L): 134 K-gluconate, 6 NaCl, 1 MgATP, and 10 HEPES (pH 7.2). The extracellular solution included (in mmol/L): 137 NaCl, 5.4 KCl, 2 CaCl_2_, 1 MgSO_4_, 10 HEPES, and 10 glucose (pH 7.3).

#### L-Type Calcium current (I_Ca, L_​) recordings

L-type Calcium currents were recorded in the whole-cell configuration using voltage-clamp. From a holding potential of −80 mV, a 400 ms ramp to −40 mV suppressed I_*Na*_​, followed by 100 ms test pulses from − 60 mV to + 60 mV in 10 mV increments (0.5 Hz). The intracellular solution contained (in mmol/L): 0.02 EGTA, 0.1 GTP-Tris, 10 HEPES, 92 K-aspartate, 48 KCl, 1 Mg-ATP, and 4 Na_2_-ATP (pH 7.2). The extracellular solution comprised (in mmol/L): 2 CaCl_2_, 10 glucose, 10 HEPES, 4 KCl, 1 MgCl_2_, 140 NaCl, and 2 probenecid (pH 7.4). Potassium currents were blocked with 5 mmol/L 4-aminopyridine and 0.1 mmol/L BaCl_2_.

#### Potassium current (I_K_) recordings

Potassium currents were recorded in voltage-clamp mode. From a holding potential of −80 mV, 500 ms depolarizing steps (−60 mV to + 60 mV in 10 mV increments) were applied, preceded by a 10 ms prepulse to −40 mV to inactivate I_*Na*_ ​. A 100 ms hyperpolarizing pulse to −120 mV followed each depolarizing step to assess inward rectifier activity. The protocol quantified transient, sustained, and inward rectifying I_*K*_ ​ currents.

#### Data quality control

Only recordings with stable access resistance and leak currents below 10% of the peak current amplitude were included in the analysis. Data were normalized to cell capacitance.

### Seahorse metabolic assay

NRVCMs were seeded in collagen-coated Agilent Seahorse 96-well plates. After 72 h of culture, the Mito Stress Test was performed using inhibitors (Oligomycin, FCCP, Rotenone/Antimycin A) prepared at 10x final concentrations. ATP rate assay measured oxygen consumption and extracellular acidification rates. Glycolytic rate assay and Mito fuel flex assay was performed as per the manufacturer’s instructions for glycolysis mediated energy measurements. AMCMs were plated in laminin coated 96-well seahorse plates and assay was conducted 36hrs post infection. Results were normalized to cell density and analyzed with Seahorse and GraphPad Prism software.

### Mitochondria isolation

Mitochondria were isolated using the Mitochondria Isolation Kit (Thermo Fisher). Cell lysates were processed sequentially with reagents A, B, and C as per the manufacturer’s protocol, followed by centrifugation at 12,000 x g. The mitochondrial pellet was resuspended and protein concentration measured using the Bio-Rad DC Assay.

### Contractility assay

NRVCMs were stained with 200 nM TMRM dye 24 h prior to the contractility assay. Fast imaging (1000 frames/30 seconds) was performed using a Zeiss Axio Observer microscope. Image analysis was conducted using Python and MATLAB algorithms, followed by statistical analysis against controls.

### Statistical analysis

All results shown are the means ± SEM. The statistical analyses of the data were performed using a two-tailed Student’s *t-*test for every experimental analysis. If necessary, one- or two-way ANOVA (followed by Student-Newman-Keuls post hoc tests when appropriate) was applied. *p*-values of less than 0.05 were considered statistically significant for all experiments.

## Results

### Letm1 is upregulated in ischemic heart and dysregulates mitochondrial pathways in cultured cardiomyocytes

**Fig. 1:**
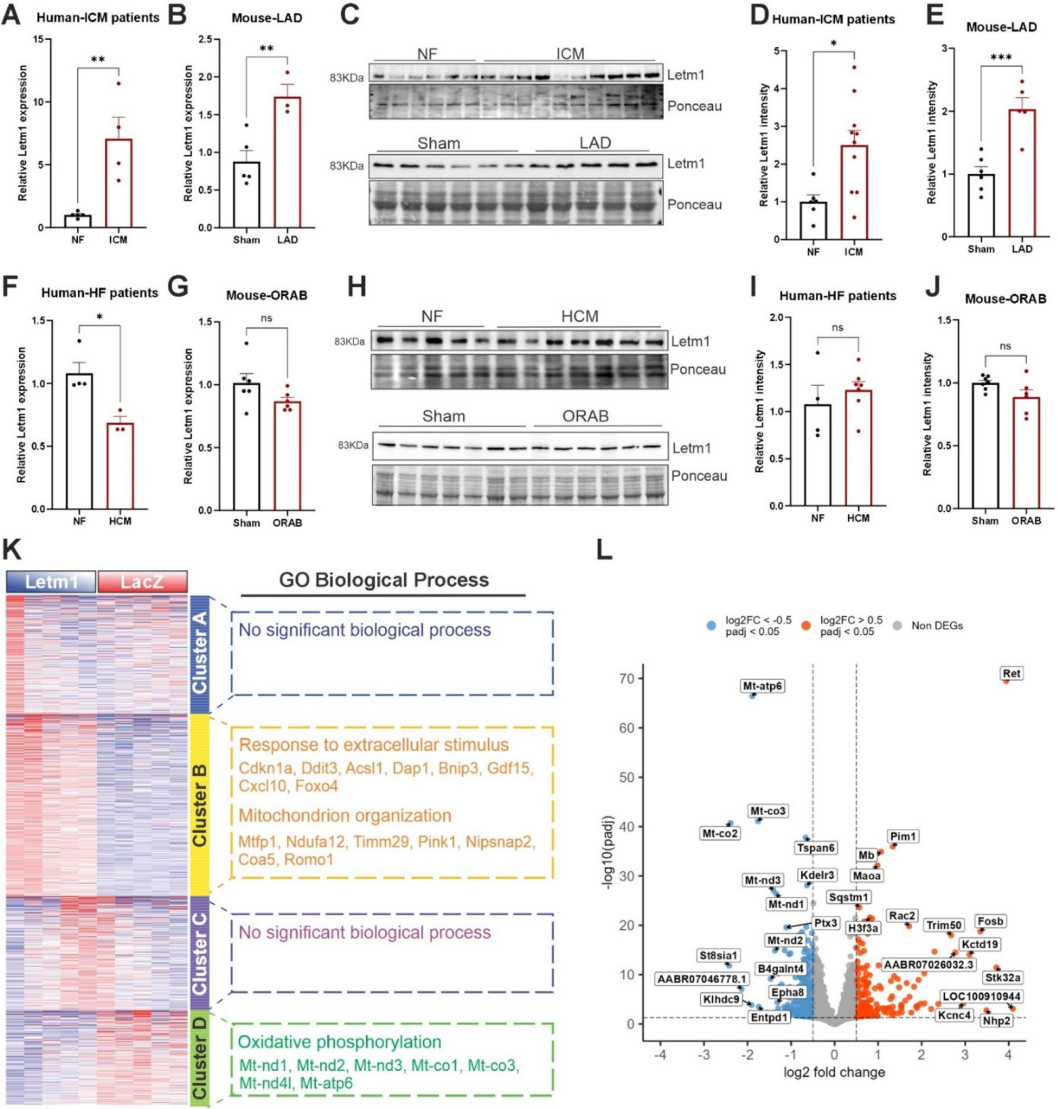
Letm1 is upregulated in ischemic heart and dysregulates mitochondrial pathways in cultured cardiomyocytes. **A**. Graph showing transcript levels of ventricular Letm1 in human patients (*n* = 5 and ICM *n* = 4) (**B**) and mouse model (Sham *n* = 5, LAD *n* = 3) of ischemic cardiomyopathy compared to respective non-failing hearts. **C**. Protein levels of ventricular Letm1 in human patients (NF *n* = 5, ICM *n* = 10) and mouse model of ischemic cardiomyopathy (Sham *n* = 6, LAD *n* = 5) compared to respective non-failing hearts. Its densitometry is depicted in **D** and **E**, respectively. Transcript levels of ventricular Letm1 in human patients (NF *n* = 4, HCM *n* = 3) (**F**) and mouse model (*n* = 6 ORAB *n* = 6) (**G**) of cardiac hypertrophy compared to respective non-failing hearts. **H**. Protein levels of ventricular Letm1 in human patient and mouse model of cardiac hypertrophy compared to respective non-failing hearts(NF *n* = 5, HCM *n* = 7, Sham *n* = 7, ORAB *n* = 6). Its densitometry is depicted in **I** and **J**, respectively. **K**. Heatmap of differentially expressed genes upon Letm1 expression compared to LacZ control. **L**. Volcano plot highlights some of the significantly up- and down-regulated genes upon Letm1 expression compared to LacZ control. NF: non-failing; HCM: hypertrophic cardiomyopathy; ICM: Ischemic cardiomyopathy; LAD: left anterior descending artery ligation; ORAB: O-ring aortic bandin; GO: Gene ontology, Statistical significance is calculated by two-tailed Students’ t-test. *, *p* < 0.05; **, *p* < 0.01; ***, *p* < 0.001; ns, non-significant

Letm1, a mitochondrial inner-membrane protein, was initially identified as a gene associated with the Wolf-Hirschhorn Syndrome (WHS), linked to the deletion of the short arm of chromosome 4 [19]. Letm1 expression was observed across multiple tissues, including the heart (Supplementary Fig. 1A), its cardiac role however is yet unclear. As previously demonstrated, co-immunostaining of Letm1 with Mitotracker confirmed its mitochondrial localization in isolated adult mouse cardiomyocytes (Supplementary Fig. 1B). To evaluate the clinical relevance in cardiac pathophysiology, we determined the expression of Letm1 in the hearts of human patients and mouse models of cardiac ischemia and heart failure. Expression analyses revealed that Letm1 transcript levels were significantly downregulated in human cardiac samples from patients with heart failure (HF) due to cardiac hypertrophy (HCM) but significantly upregulated in ischemic cardiomyopathy (ICM) (Fig. 1A, F). Similar regulation was observed in murine models- Letm1 levels increased after cardiac ischemia induced by left anterior descending (LAD) artery ligation but decreased (non-significant) after cardiac hypertrophy due to pressure overload induced by O-ring aortic banding (ORAB) (Fig. 1B, G). Although Letm1 protein level was upregulated in ICM patients and LAD-ligated mouse hearts, it remained unchanged in HCM patients and ORAB mouse hearts compared to the respective controls (Fig. 1C-E and H-J). Furthermore, we also observed significant upregulation of Letm1 in cultured neonatal rat ventricular cardiomyocytes (NRVCMs) upon hypoxia treatment (Supplementary Fig. 1C, D). Due to the striking and consistent upregulation observed in human patients, mice and in vitro cell culture models of cardiac ischemia, both at the transcript and the protein level, we focused in this study on demonstrating the gain-of-function effects of Letm1 using the cell autonomous model of NRVCMs supplemented by AMCMs and iPSCs.

To understand the transcriptional changes upon Letm1 expression, we performed bulk RNA sequencing of NRVCMs overexpressing Letm1 in comparison with NRVCMs expressing LacZ as a control. We identified four clusters among differentially expressed genes (Figs. 1K). Interestingly, the major affected pathways were mitochondria associated processes, exhibiting significant downregulation of mitochondrial oxidative phosphorylation (OXPHOS) in cluster D (e.g. Mt-nd1, Mt-nd2, Mt-nd3, Mt-co1), and upregulation of mitochondria organization pathway (e.g. Mtfp1, Ndufa12, Timm29, Pink1, Nipsnap2, Coa5) in cluster B (Figs. 1K, L, Supplementary Fig. 1E). Moreover, genes involved in response to extracellular stimuli such as Gdf15, Cxcl10, Bnip3, Cdkn1, etc. were also significantly upregulated in NRVCMs after Letm1 overexpression in cluster B (Fig. 1K, Supplementary Fig. 1F). Cluster A and C did not align to any major gene ontology (GO) process. To further elucidate the molecular consequences of Letm1 overexpression, we performed proteomic analysis of neonatal rat ventricular cardiomyocytes (NRVCMs) under Letm1-overexpressing versus control conditions. GO enrichment revealed distinct pathway alterations in response to Letm1 elevation (Supplementary Fig. 1G-I). Notably, key pathways essential for cardiomyocyte structure and function, including actin cytoskeleton organization, phagosome signaling, calcium signaling, dilated cardiomyopathy, and striated muscle contraction, were significantly modulated. These proteomic findings, together with our transcriptomic data, suggest that Letm1 overexpression perturbs mitochondrial homeostasis, with potential implications for disrupted structure, energy metabolism and altered ion handling in cardiomyocytes.

### Letm1 dysregulates OXPHOS gene expression and protein abundance in cultured cardiomyocytes

**Fig. 2:**
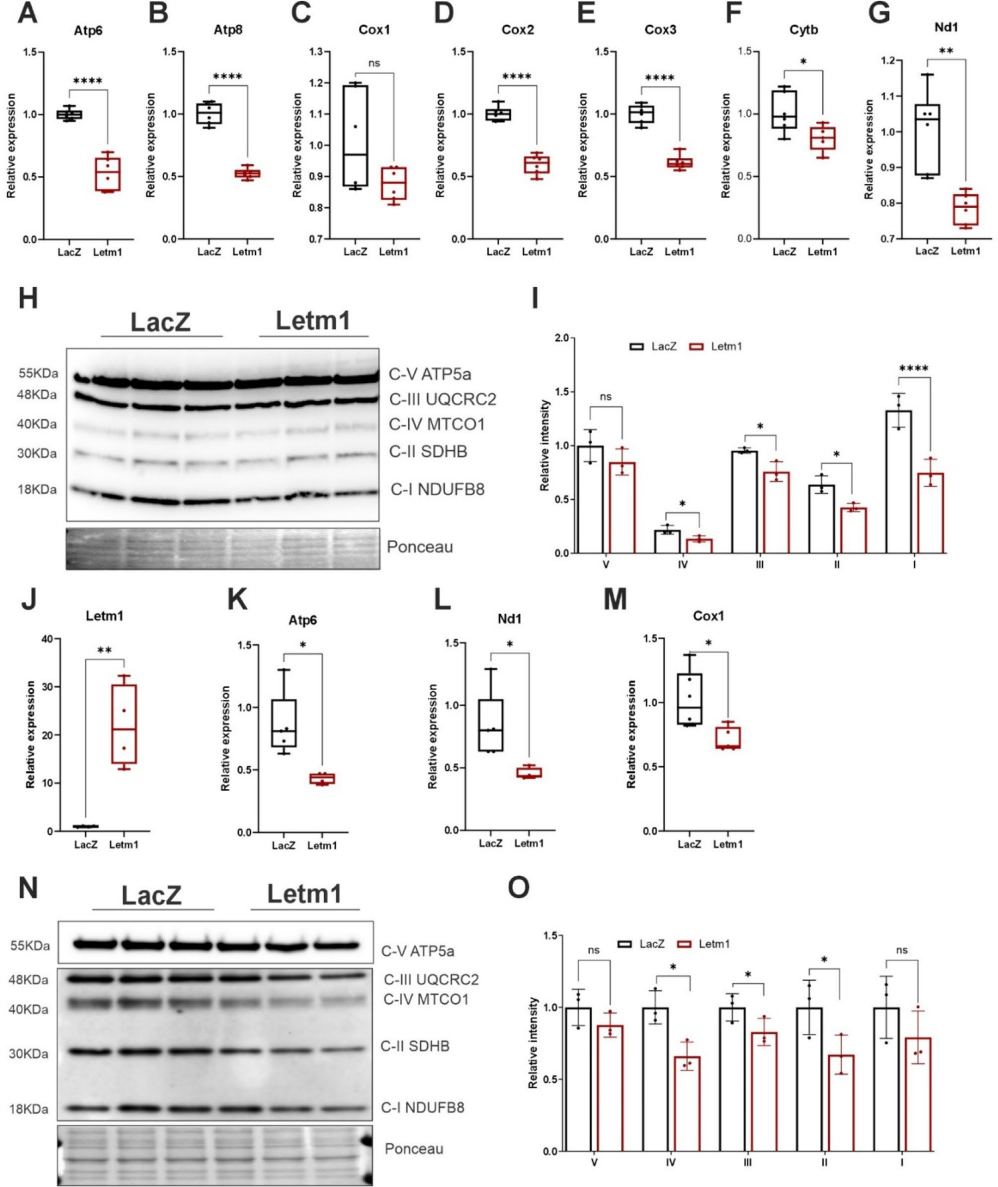
Letm1 dysregulates OXPHOS gene expression and protein abundance in cultured cardiomyocytes. **A**-**G**. Transcript level detection of genes involved in oxidative phosphorylation complexes upon Letm1 expression. **H**. Immunoblot of oxidative phosphorylation (OXPHOS) complexes detected in total protein lysates and its desitometric analysis in (**I**). **J**-**M** Transcript level alteration of mitochondrial genes in iPS derived cardiomyocytes post elevated letm1 levels as compared to LacZ control. **N**. Immunoblot of oxidative phosphorylation (OXPHOS) complexes detected in total protein lysates from iPSC-derived cardiomyocytes and its densitometric analysis in (**O**). Statistical significance is calculated by two-tailed Students’ t-test. *, *p* < 0.05; **, *p* < 0.01; ***, *p* < 0.001; ****, *p* < 0.0001; ns, non-significant

Subsequently, to experimentally validate the transcriptomics findings and to assess the impact of Letm1 elevation on mitochondria function, Letm1 was overexpressed in NRVCMs, followed by assessments using qPCR and immunoblotting. In line with the RNA-seq results, Letm1 overexpression resulted in a significant downregulation of genes encoding core components of the OXPHOS system, including Cox1, Cox2, Cox3, Nd1, Cytb, Atp8 and Atp6 (Fig. 2A-G). These genes are indispensable for the assembly and function of the electron transport chain (ETC), indicating a disruption in mitochondrial biogenesis induced by Letm1 expression. Immunoblot analyses further substantiated these findings, showing a marked reduction in OXPHOS complex protein levels in total cellular lysates, with an even greater depletion observed in isolated mitochondrial fractions (Fig. 2H-I, Supplementary Fig. 2A-B). To determine whether these effects extend beyond neonatal cardiomyocytes and are conserved across species, we overexpressed Letm1 in human iPSC-derived cardiomyocytes. Remarkably, similar transcriptomic alterations were observed in iPSC derived cardiomyocytes (Fig. 2J-M), which were also reflected at the protein level (Fig. 2N-O).

Given that Letm1 expression was previously shown to be dysregulated in hypoxic conditions and elevated in ischemic heart tissue, we next examined the effects of Letm1 overexpression under hypoxia. Consistent with a stress-exacerbated phenotype, the downregulation of OXPHOS gene expression was further amplified under hypoxic conditions (Supplementary Fig. 2C-F), suggesting that Letm1 overexpression sensitizes cardiomyocytes to metabolic dysfunction in pathological states.

Collectively, these results demonstrate that Letm1 overexpression impairs mitochondrial gene expression and disrupts the functional composition of the electron transport chain in both neonatal and human iPSC-derived cardiomyocytes, revealing a conserved susceptibility of mitochondrial homeostasis to Letm1 dysregulation across developmental stages and species.

### Letm1 overexpression impairs mitochondrial bioenergetics and functional integrity in cardiomyocytes

**Fig. 3:**
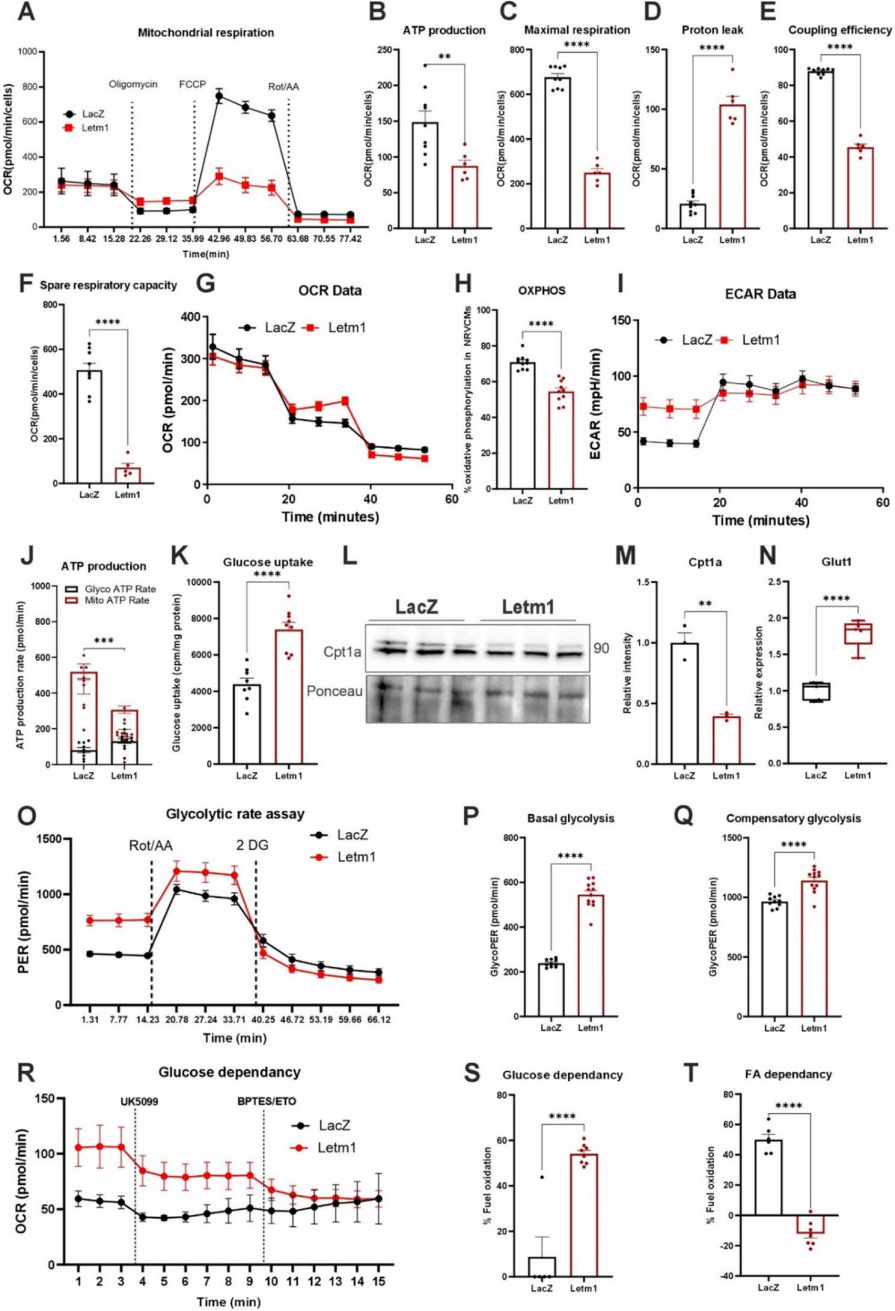
Letm1 overexpression impairs mitochondrial bioenergetics and functional integrity in cardiomyocytes. **A**. Results from mitochondrial function assessment using seahorse metabolic assay (Mito Stress Test) performed in NRVCMs indicating oxygen consumption rate (OCR) for mitochondrial respiration (**B**) ATP production, (**C**) maximal respiration, (**D**) proton leak, and (**E**) coupling efficiency (**F**). Spare respiratory capacity, **G**. Oxygen consumption rate in the mito stress test assay. **H**. Oxidative phosphorylation calculated from the ATP rate assay detecting reduced levels upon Letm1 elevation, **I**. ECAR upon Letm1 elevation, **J**. ATP production graph plotted using measurements from ATP rate assay to calculate ATP obtained from mitochondrial vs. glycolysis pathway. **K**. Graph showing the results from glucose uptake assay for NRVCMs either expressing Letm1 or LacZ control. Immunoblot (**L**) and its densitometry (**M**) depicting Cpt1a levels in NRVCMs expressing Letm1 compared to LacZ control. **N**. Graph showing transcript levels of Glut1 in NRVCMs expressing Letm1 compared to LacZ control. **O**. Proton efflux rate from the glycolytic rate assay upon Letm1 overexpression. **P**. Elevated levels of basal glycolysis upon Letm1 overexpression as compared to control. **Q**. Increase in compensatory glycolysis upon Letm1 overexpression. **R**. Substrate utilization assay revealed glucose dependency as the main substrate utilized for ATP generation in Letm1 overexpressing cells. **S**. Increased % of glucose dependency of Letm1 overexpressing cells as compared to LacZ condition. **T**. Decreased % of fatty acid utilization of Letm1 overexpressing cells as compared to LacZ condition. ECAR; Extracellular acidification rate, OCR; oxygen consumption rate, PER; proton efflux rate. *n* = 3 for all experiments. Statistical significance is calculated by two-tailed Students’ t-test. *, *p* < 0.05; **, *p* < 0.01; ***, *p* < 0.001; ****, *p* < 0.0001; ns, non-significant

To assess whether Letm1 overexpression leads to mitochondrial dysfunction, we performed Seahorse metabolic analyses. Cardiomyocytes with elevated Letm1 showed reduced ATP production and maximal respiratory capacity, along with increased proton leak, indicative of mitochondrial stress (Fig. 3A-D). Although basal respiration remained largely unaffected, both coupling efficiency and spare respiratory capacity were significantly compromised, pointing to diminished mitochondrial performance (Fig. 3E-F). Consistent with this, ATP rate assays showed a sharp drop in mitochondrial ATP output and a rise in extracellular acidification, suggesting a metabolic shift from oxidative phosphorylation to glycolysis (Fig. 3G-I). Quantification of ATP sources confirmed this switch, with Letm1-overexpressing neonatal cardiomyocytes producing less mitochondrial ATP and more glycolytic ATP (Fig. 3J). Importantly, these effects were also observed in adult mouse cardiomyocytes, which exhibited similar reductions in mitochondrial ATP and increased glycolytic reliance, underscoring the broader relevance of this metabolic shift (Supplementary Fig. 3A-D).

Letm1-overexpressing cells also showed increased glucose uptake, downregulation of Cpt1a (involved in fatty acid oxidation), and upregulation of Glut1 (a glucose transporter), consistent with a glycolytic shift (Fig. 3K-N). Glycolytic rate assays further confirmed elevated glycolytic activity (Fig. 3O-Q). Fuel flexibility tests revealed a stronger dependence on glucose substrate utilization and reduced fatty acid oxidation (Fig. 3R-T). In summary, Letm1 overexpression induced robust metabolic reprogramming in both neonatal and adult cardiomyocytes, marked by impaired mitochondrial respiration, reduced ATP output, and increased reliance on glycolysis.

### Elevated levels of Letm1 disrupts electrophysiology and contractility in cardiomyocytes

**Fig. 4:**
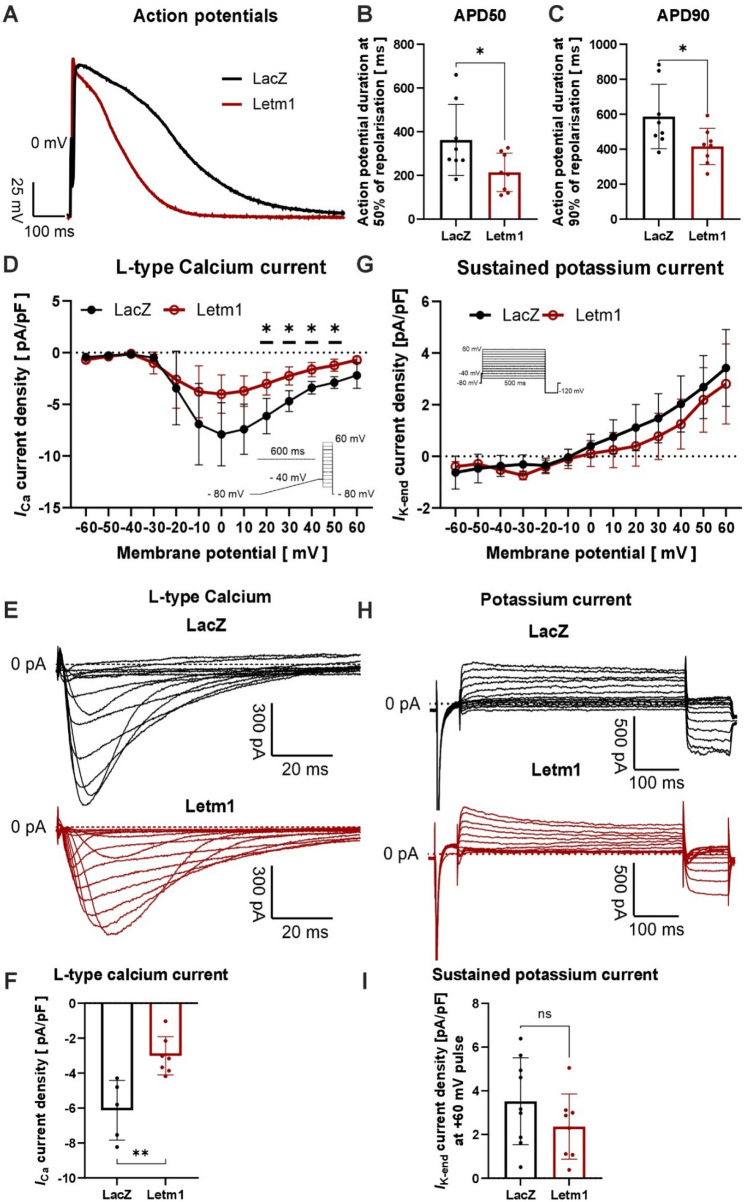
Elevated levels of Letm1 disrupts electrophysiology and contractility in cardiomyocytes. **A** Representative graphs of action potential measurements of cardiomyocytes expressing Letm1 as compared to LacZ control. **B** Graph plotted for action potential duration at 50% (**B**) and 90% (**C**) repolarization. **D**. L-type Calcium current density of cells with Letm1 vs. LacZ. **E**. Representative Calcium current measurements of Letm1 vs. LacZ expressing cells. **F**. Calculated Calcium current density. **G**. Sustained Potassium current of cells with Letm1 vs. LacZ. **H**. Representative sustained Potassium current measurements of Letm1 vs. LacZ expressing cells. **I**. Calculated Potassium current density. APD: action potential duration, *n* = 3. Statistical significance is calculated by two-tailed Students’ t-test. *, *p* < 0.05; **, *p* < 0.01; ns, non-significant

**Fig. 5:**
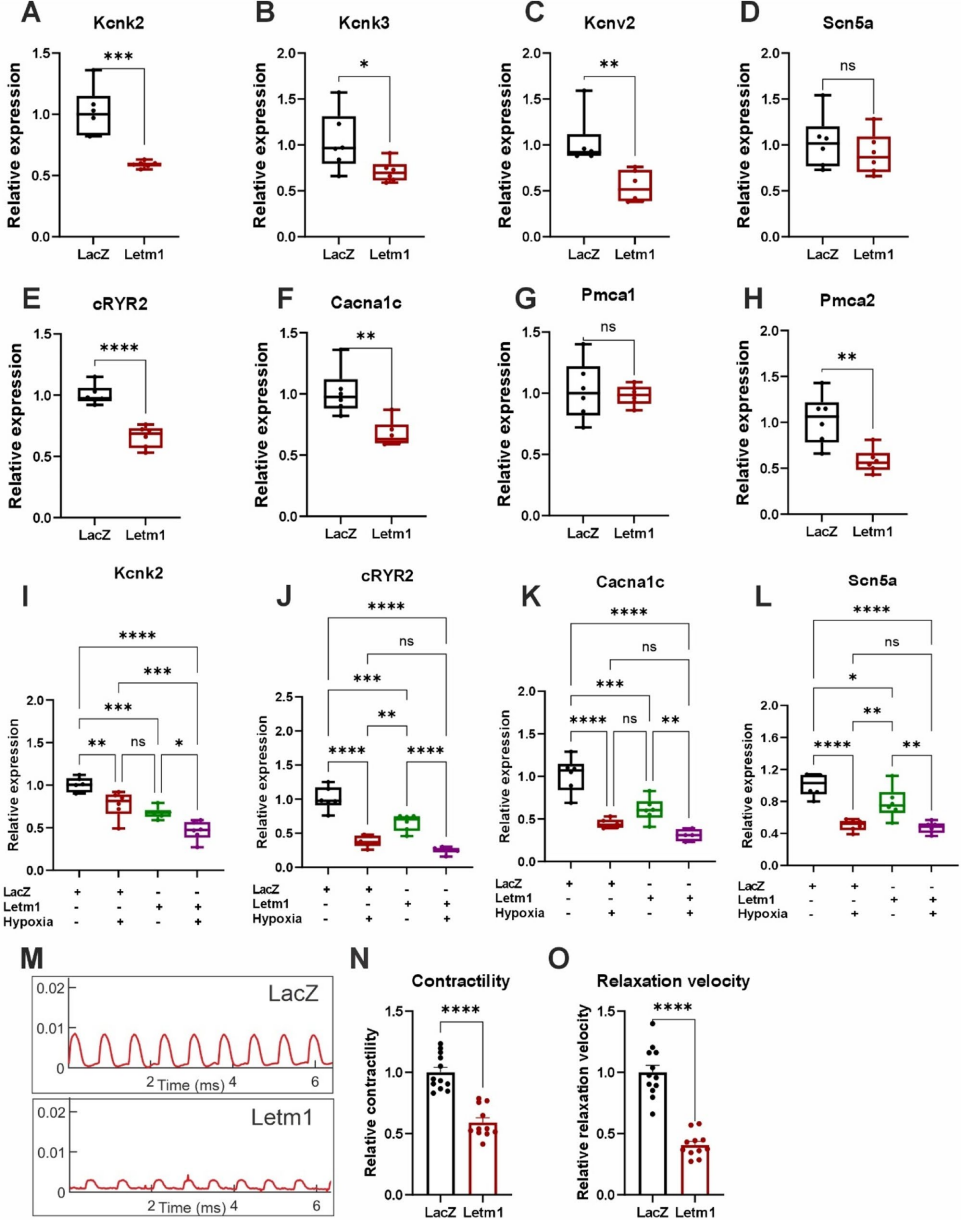
Letm1 expression disrupts contractility in cardiomyocytes. **A**-**H**. Transcript levels of genes involved in Potassium, sodium and Calcium ion transport in Letm1 expressing or LacZ expressing control NRVCMs. **I**.-**L**. Transcript levels of genes involved in Potassium, sodium and Calcium ion transport in Letm1 expressing or LacZ expressing control in NRVCMs under hypoxia condition as compared to normoxia condition. **M**. Representative images of the peaks obtained from cardiomyocyte contractility assay. **N**. Cardiomyocyte contractility calculated from the obtained data. **O**. Relaxation velocity of cardiomyocytes upon Letm1 expression. *n* = 3. Statistical significance is calculated by two-tailed Students’ t-test. *, *p* < 0.05; **, *p* < 0.01; ***, *p* < 0.001; ****, *p* < 0.0001; ns, non-significant

Given the debatable role of Letm1 as a Potassium, Calcium, and proton exchanger, as well as the altered calcium signaling pathway in proteomics (Supplementary Fig. 1H-I) we investigated whether its overexpression impacts calcium and/or potassium ion transport and electrophysiological properties in cardiomyocytes. Using NRVCMs overexpressing Letm1, we conducted patch-clamp analyses to assess action potential dynamics, with control cells expressing LacZ as reference group. Letm1 overexpression resulted in significant shortening of the action potential duration at both 50% (APD50) (with an SEM of −141.9 ± 57.10 and *p* = 0.02) and 90% (APD90) (with an SEM of −144.4 ± 66.72 and *p* = 0.04) repolarization, indicating pronounced alterations in repolarization dynamics (Fig. 4A-C). Interestingly, key parameters such as cell surface area, action potential amplitude, resting membrane potential and upstroke velocity remained unaffected, suggesting that Letm1 specifically impacts repolarization mechanisms without broadly impairing cellular excitability (Supplementary Fig. 4A-D). Since repolarization is intricately linked to calcium ion flux, we next evaluated L-type calcium currents in NRVCMs. Letm1-overexpressing cells exhibited a significant reduction in L-type calcium current density, underscoring defects in calcium handling as a potential driver of the observed electrophysiological abnormalities (Fig. 4D-F). Interestingly however, we did not observe any significant effect of Letm1 overexpression on sustained potassium current (Fig. 4G-I, Supplementary Fig. 4E, F). Further analysis of ion homeostasis revealed dysregulated transcript levels of genes involved in potassium and calcium transport (Fig. 5A-H). Consistent with a stress-mediated phenotype, the downregulation of ion homeostasis gene expression was further exacerbated under hypoxic conditions (Fig. 5I-L). These transcriptional changes likely underscore the molecular basis of Letm1-mediated calcium-ion transport disruption.

Given the integral role of calcium flux in driving cardiomyocyte contractility, we next evaluated whether Letm1 overexpression affects contractile function. Contractility assays revealed a marked reduction in contraction and relaxation velocities, indicating impaired cardiomyocyte contractility (Fig. 5M-O). Additionally, Letm1-overexpressing cells displayed an elevated beat rate that inversely correlated with contractile performance (Supplementary Fig. 5A, B). Under healthy conditions, contraction strength and beat rate are typically aligned; the observed inverse relationship suggest a shift towards a diseased phenotype, likely reflective of underlying cardiac dysfunction.

Together, these findings highlight the multifaceted impact of Letm1 overexpression on cardiomyocyte physiology, encompassing profound disruptions in ion transport, electrophysiology, and contractile function. These results provide critical mechanistic insights into the role of Letm1 in cardiomyocyte pathophysiology and its potential contribution to cardiac disease progression.

### Letm1 overexpression alters mitochondrial ultrastructure and sarcomeric organization in adult cardiomyocytes

**Fig. 6:**
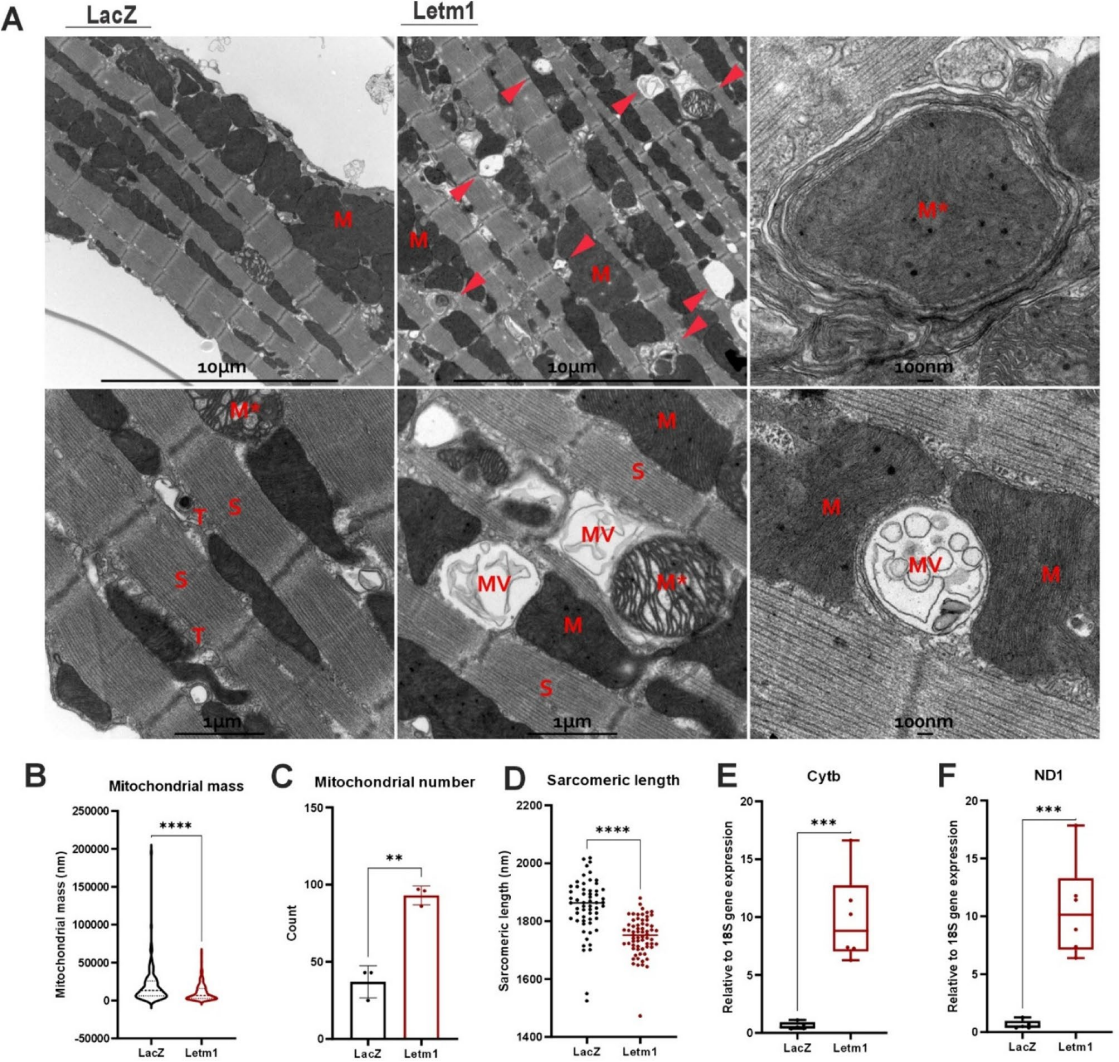
Letm1 overexpression alters mitochondrial ultrastructure and sarcomeric organization in adult cardiomyocytes. **A** Representative electron microscopy images of Letm1 overexpressing adult cardiomyocytes as compared to LacZ expressing control. In overviews (10 μm scale), cardiomyocytes with Letm-1 overexpression feature large vacuoles typically at the Z-level of sarcomeres (arrowheads). Images at higher magnification (1 μm scale) demonstrate disturbance of the Membrane-system in Letm1 condition as compared to LacZ control (T, sarcoplasmic T-tubule system), containing multivesicles (MV) and cellular content, including mitochondria (M), reminiscent of autophagosomes. The detailed view (100 nm scale) in the upper panel depicts defective mitochondria inside a vesicle (M*) and in the lower panel healthy mitochondria and multivesicular body (MV) between two regular mitochondria (M). S; sarcomere, M; mitochondria, M*; defective mitochondria, T; T tubules. **B** Reduced mitochondrial mass was observed in Letm1 overexpressing cells as compared to control cell. **C** Mitochondrial number was observed to be increased in count upon Letm1 overexpression. **D** Sarcomeric length was significantly reduced in cells overexpressing Letm1, **E**, **F**. Quantitative mitochondrial DNA copy number was determined using realtime PCR of mitochondrial to nuclear genes at DNA level. *n* = 3. Statistical significance is calculated by two-tailed Students’ t-test. *, *p* < 0.05; **, *p* < 0.01; ***, *p* < 0.001; ****, *p* < 0.0001; ns, non-significant

Given the proteomic evidence of disrupted sarcomeric and cytoskeletal assembly (Supplementary Fig. 1H, I), we examined whether Letm1 overexpression affects cardiomyocyte ultrastructure. Transmission electron microscopy (TEM) imaging of adult mouse cardiomyocytes revealed clear structural abnormalities, including large vacuoles near sarcomeric Z-discs and disrupted vesicular membranes (Fig. 6A, arrowheads). We also observed multivesicular bodies (MVBs) containing mitochondrial fragments and autophagosome-like structures, indicating active mitophagy. High-resolution images further showed damaged mitochondria (M*) and mitophagic intermediates, consistent with enhanced mitochondrial turnover. Quantitative analysis showed a decrease in mitochondrial mass but an increase in mitochondrial number and reduced sarcomere length in Letm1-overexpressing cells (Fig. 6B-D), suggesting mitochondrial fragmentation alongside compensatory biogenesis and cytoskeletal destabilization.

To confirm biogenesis, we measured mitochondrial DNA (mtDNA) content. Both adult and neonatal Letm1-overexpressing cardiomyocytes exhibited significantly elevated mtDNA copy number (Fig. 6E-F, Supplementary Fig. 6A-B). This increase occurred despite the downregulation of OXPHOS genes and proteins, likely indicating a structural, but not functional, compensatory response to mitochondrial stress. Overall, Letm1 overexpression led to extensive ultrastructural remodeling in cardiomyocytes, marked by mitochondrial fragmentation, increased mitophagy, and sarcomeric disruption.

### Letm1 overexpression activates autophagy but impairs lysosomal clearance, leading to apoptotic cell death in cardiomyocytes

**Fig. 7:**
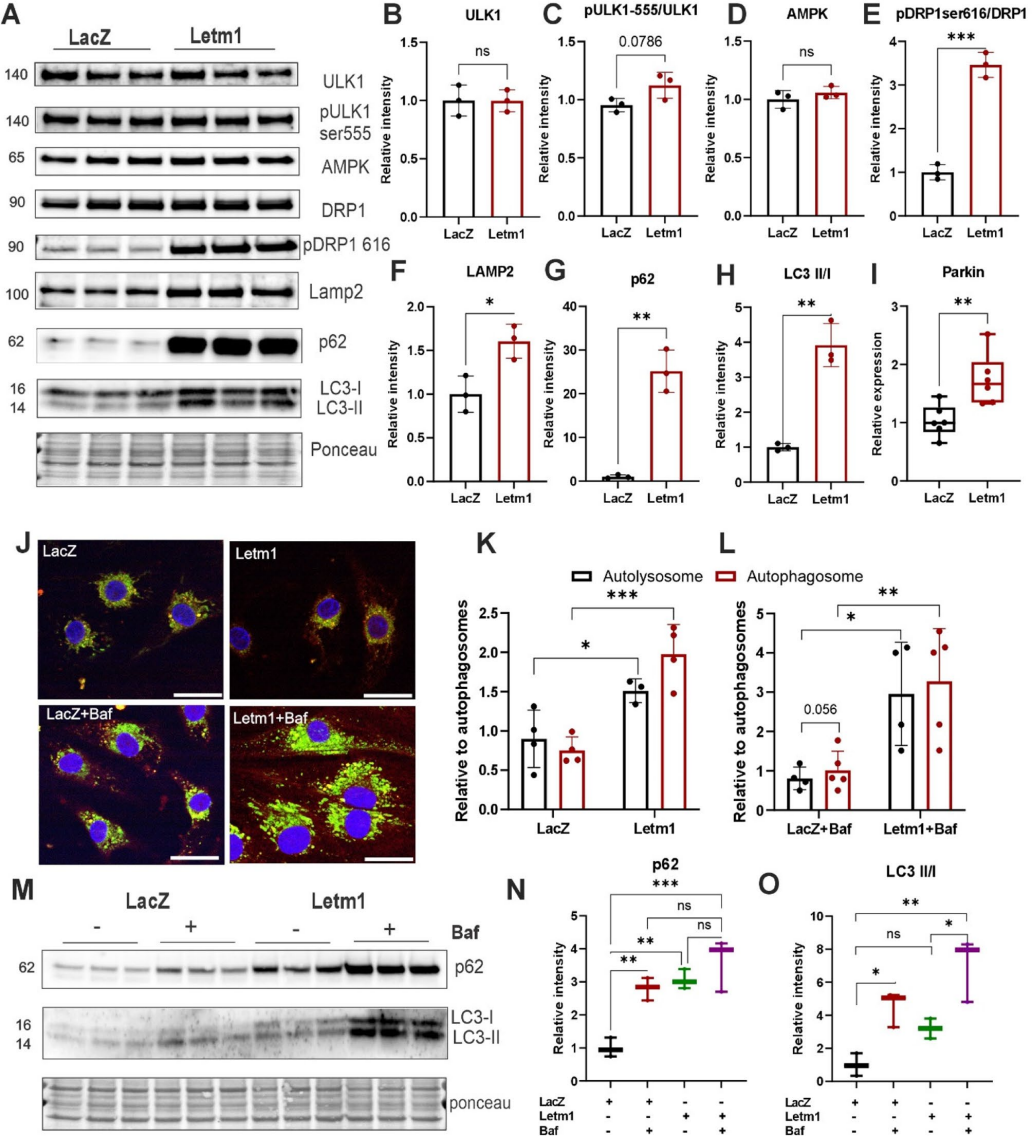
Letm1 overexpression activates autophagy but impairs lysosomal clearance. **A**. Immunoblots indicating different markers for AMPK-ULK1 signaling pathway and autophagy in Letm1 overexpressing NRVCMs as compared to LacZ control condition along with their respective densitometric analysis in (**B**-**H**). **I**. Increased transcript level of Parkin upon Letm1 elevation. **J**. Representative images of Tandem LC3-GFP-RFP imaging in cells with increased Letm1 in combination with BafilomycinA1 treatment and its respective analysis in **K**, **L**. **M**. Immunoblots indicating p62 and LC3 levels upon Letm1 overexpression as compared to LacZ control in presence and absence of Bafilomycin A1 treatment and its respective densitometric analysis in **N**, **O** respectively. Baf; BafilomycinA1. *n* = 3. Statistical significance is calculated by two-tailed Students’ t-test or 2-way ANOVA. *, *p* < 0.05; **, *p* < 0.01; ***, *p* < 0.001; ****, *p* < 0.0001; ns, non-significant

Given the extensive structural and metabolic changes observed with Letm1 overexpression, we next examined its impact on key regulators of mitochondrial dynamics and autophagy pathways flagged as disrupted in our proteomic data (Supplementary Fig. 1H, I). Both in neonatal and adult cardiomyocytes, Letm1 overexpression significantly increased total ULK1 and its activating phosphorylation at Ser555, alongside elevated AMPK levels (Fig. 7A-D, Supplementary Fig. 7A-C), indicating energy stress and autophagy initiation. Letm1 also upregulated DRP1 expression and Ser616 phosphorylation (Fig. 7A, E, Supplementary Fig. 7A, D), suggesting enhanced mitochondrial fission, which aligns with the increased mitochondrial number and fragmentation seen in previous ultrastructural analyses (Fig. 6). Furthermore, elevated LAMP2 levels (Fig. 7A, F) suggested increased lysosomal content. However, despite activation of upstream autophagy genes/molecules, autophagic flux appeared impaired: both by p62 and LC3 accumulation (Fig. 7A, G, H; Supplementary Fig. 7A, E, F), indicating defective autophagosome clearance.

Parkin1 transcript levels were also upregulated (Fig. 7I; Supplementary Fig. 7G), pointing to activation of PINK1-Parkin-mediated mitophagy. Yet, LC3-GFP-RFP reporter assay revealed accumulation of yellow puncta, reflecting stalled autophagosomes that failed to fuse with acidic lysosomes (Fig. 7J, K; Supplementary Fig. 7H, I). This block was confirmed and amplified by Bafilomycin A1, a lysosomal acidification inhibitor, which further increased LC3-II and p62 levels (Fig. 7L-O), reinforcing a defect in autophagosome degradation rather than enhanced initiation alone. Expression of PGC-1α, PPARα, ERRα, and NRF1, key transcriptional drivers of mitochondrial biogenesis, were also elevated (Supplementary Fig. 7J), indicating an attempted compensatory response to mitochondrial stress and dysfunction. The disconnect between autophagy activation and lysosomal degradation suggested a failure in flux resolution, consistent with the observed accumulation of autophagic intermediates and vacuoles under TEM. These defects were also present in adult cardiomyocytes, underscoring the translational robustness of the phenotype.

**Fig. 8:**
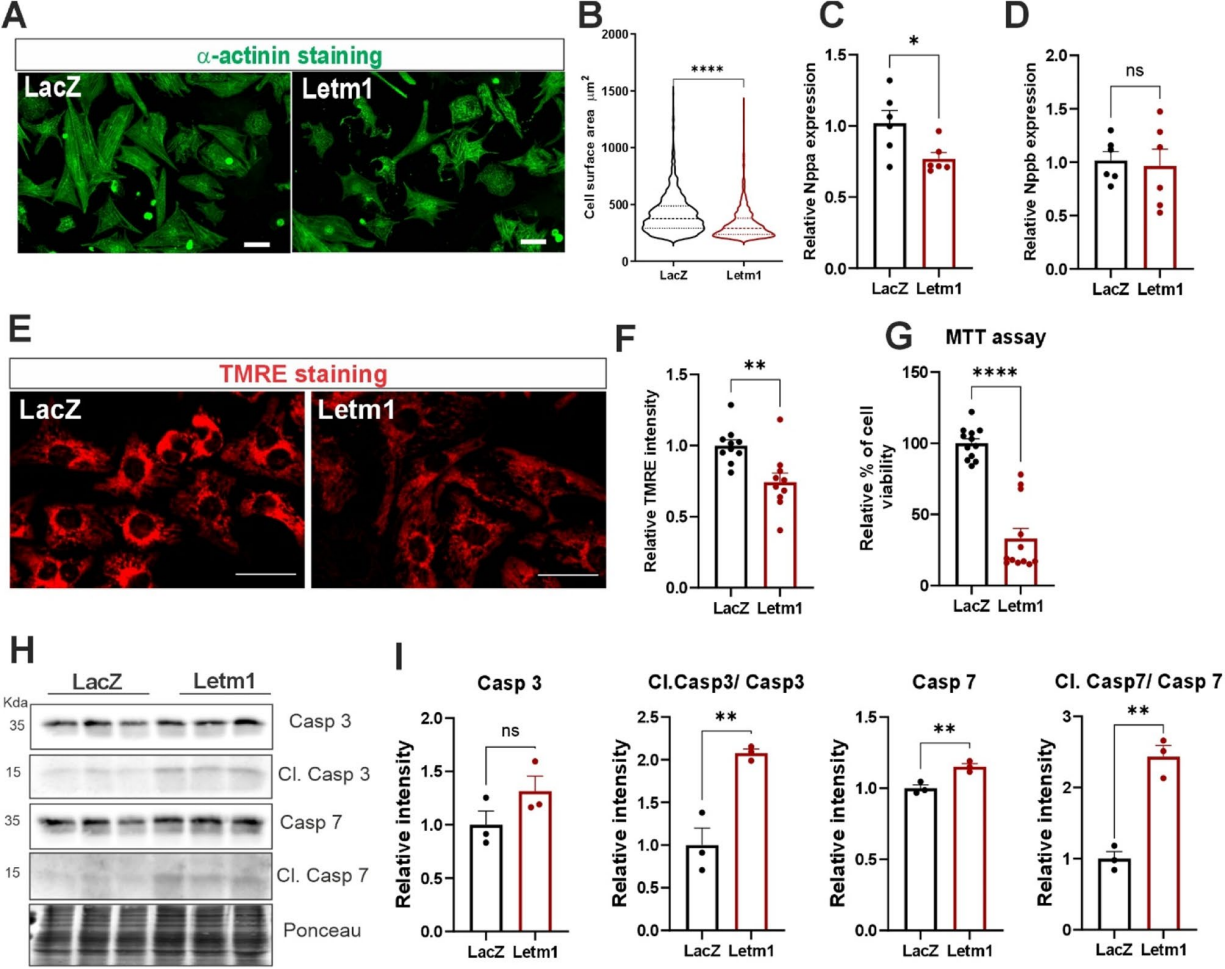
Letm1 overexpression impairs cardiomyocyte survival, and stress response via mitochondrial dysfunction and increased apoptosis. **A**. Representative immunofluorescence images of cardiomyocytes stained with α-actinin and DAPI (scalebar 50 μm) for cell surface area measurement and it analysis is shown in (**B**). Transcript levels of Nppa (**C**) and Nppb (**D**), in cardiomyocytes with either Letm1 or LacZ overexpression. **E**. Representative immunofluorescence images of cardiomyocytes stained with TMRE (Scalebar 50 μm) for mitochondrial membrane potential measurements depicted in (**F**). **G**. Cardiomyocyte cell viability detected by MTT assay. **H**. Immunoblots detecting Caspase 3 and its cleaved fragment as well as Caspase 7 and its cleaved fragment. Its densitometry measurements are depicted in **I**, respectively. *n* = 3 for every experiment. Statistical significance is calculated by two-tailed Students’ t-test. *, *p* < 0.05; **, *p* < 0.01; ***, *p* < 0.001; ****, *p* < 0.0001; ns, non-significant

To assess downstream effects on cell viability, Letm1-overexpressing cardiomyocytes showed reduced surface area and Nppa expression, while Nppb remained unchanged (Fig. 8A-D). TMRE staining revealed a significant drop in mitochondrial membrane potential (ΔΨm), consistent with impaired mitochondrial function and increased oxidative stress, as evidenced by higher MitoSOX signal (Fig. 8E, F; Supplementary Fig. 8A). Cell viability and numbers were also decreased (Fig. 8A, G), and caspase activation confirmed apoptosis (Fig. 8H). These effects were consistently observed in neonatal, adult and human iPSC-derived cardiomyocytes (Supplementary Fig. 8B-G).

## Discussion

Letm1 is historically recognized as a mitochondrial potassium-proton exchanger implicated in Wolf-Hirschhorn Syndrome. Previous studies have reported its critical role in mitochondrial ion exchange, but its involvement in cardiac disease has been largely unexplored. Our studies identify a pivotal role of Letm1 in cardiomyocyte function and survival, uncovering its deleterious effects on mitochondrial bioenergetics, ion homeostasis, and contractility. Using a comprehensive approach that integrates transcriptomics, proteomics, biochemical assays, and functional studies, we demonstrate that Letm1 upregulation profoundly impacts cardiomyocyte physiology, elucidating mechanisms that could underlie its role in cardiac pathophysiology, particularly in ischemic heart disease. Our findings demonstrate for the first time that Letm1 is significantly upregulated in ischemic cardiomyopathy in both human and mouse models. This contrasts with its unaltered levels in hypertrophic heart failure, indicating a context-specific regulation of Letm1 in cardiac pathology. These observations underscore its potential as a marker of ischemic remodeling and warrant further exploration into its regulatory mechanisms during ischemia-reperfusion injury.

### Letm1 disrupts mitochondrial bioenergetics

The most striking finding of this study is the impact of Letm1 on mitochondrial bioenergetics in primary rat as well as human iPSC-derived cultured cardiomyocytes. Letm1 overexpression significantly downregulated key genes and proteins of the oxidative phosphorylation (OXPHOS) system, including those encoding subunits of complexes I (e.g., Mt-nd1), III (Mt-Cytb), IV (Mt-Cox1, Mt-Cox2, Mt-Cox3), and V (Mt-Atp6), underscoring a disruption in the electron transport chain (ETC). Functionally, these changes translated into reduced ATP production, diminished spare respiratory capacity, and heightened proton leak, all of which reflect mitochondrial stress and inefficiency [41–43].

Intriguingly, despite these profound impairments in mitochondrial oxidative metabolism, Letm1 overexpression was associated with increased glucose uptake. This observation aligned with the upregulation of Pparδ and Glut1, key regulators of glucose metabolism, observed in our transcriptomic data. Pparδ plays a central role in metabolic flexibility, enhancing glucose and fatty acid oxidation in energy-demanding tissues, including the heart. However, in the context of Letm1-mediated mitochondrial dysfunction, the upregulation of Glut1 suggests a compensatory shift toward glycolytic energy production to mitigate the ATP deficit caused by impaired oxidative phosphorylation. Such metabolic reprogramming is a hallmark of stressed or failing cardiomyocytes, as they prioritize glycolysis over mitochondrial respiration to meet energy demands under pathological conditions [44, 45]. Glut1, a critical glucose transporter, is similarly upregulated in ischemic myocardium to enhance glucose utilization when fatty acid oxidation is impaired [46]. This metabolic shift, while adaptive in the short term, may contribute to long-term cardiac dysfunction, as glycolysis is less efficient in ATP production compared to OXPHOS.

Conversely, the observed downregulation of Cpt1a, a critical regulator of fatty acid oxidation, further reinforces the suppression of mitochondrial oxidative metabolism. Cpt1a facilitates the transport of long-chain fatty acids into mitochondria for β-oxidation, a key energy source for cardiomyocytes under normal conditions [47]. The combined effects of Pparδ, Glut1, and Cpt1a dysregulation highlight a metabolic inflexibility that could underlie the bioenergetic failure in Letm1-overexpressing cardiomyocytes. This shift in substrate utilization highlights a maladaptive metabolic reprogramming that could underpin the diminished mitochondrial efficiency observed in our Seahorse metabolic analyses. Furthermore, elevated proton leak suggests mitochondrial membrane instability, which may exacerbate reactive oxygen species (ROS) production. Notably, we observed a reduction in mitochondrial membrane potential (ΔΨm), a critical determinant of mitochondrial integrity. This aligns with studies showing that ΔΨm dysregulation contributes to mitochondrial fragmentation and subsequent cardiomyocyte apoptosis during cardiac stress [48, 49]. Together, these findings suggest that Letm1 expression promotes a metabolic phenotype characterized by reduced mitochondrial bioenergetics, enhanced glucose dependency, and impaired fatty acid oxidation, consistent with a pathological remodeling of cardiomyocyte metabolism likely spiraling down to stress induced cardiomyocyte death.

### Implications for ion homeostasis, electrophysiology and contractile dysfunction

Letm1’s established role as a Potassium, Calcium, and proton exchanger places it at the nexus of ion homeostasis. Concordantly, we demonstrate in this study that in cardiomyocytes, its overexpression led to significant disruptions in L-type calcium currents, shortening of action potential duration, and impaired contraction-relaxation dynamics. These electrophysiological abnormalities are consistent with mitochondrial dysfunction and transcriptional dysregulation of calcium-handling genes such as Ryr2 and Cacna1c, which together compromise excitation-contraction coupling [50, 51]. calcium dysregulation is a hallmark of heart failure and arrhythmogenesis, as it directly impairs excitation-contraction coupling and promotes electrical instability [51]. Interestingly, despite alterations in calcium handling, potassium currents remained unaltered, highlighting the selective effects of Letm1 on specific ion channels. This specificity in Letm1’s impact on calcium channels highlights its nuanced role in modulating electrophysiological properties.

Beyond electrophysiology, Letm1 overexpression significantly impaired cardiomyocyte contractility, as evidenced by reduced contraction and relaxation velocities. The inverse relationship between contractility and beating rate observed in Letm1-expressing cells suggests a pathological shift, likely reflecting maladaptive remodeling under stress conditions. This phenomenon has been observed in models of heart failure, where increased beat rates fail to compensate for contractile deficits, exacerbating cardiac dysfunction [52]. These findings suggest that Letm1-mediated disruptions in calcium transport and mitochondrial energetics act synergistically to compromise contractile performance, as evidenced by reduced contraction and relaxation velocities.

### Effect on the ultrastructure of cardiomyocytes

In line with the pronounced mitochondrial remodeling observed at the molecular and functional level, ultrastructural analysis by electron microscopy revealed striking morphological abnormalities in adult cardiomyocytes overexpressing Letm1. Specifically, we noted a substantial increase in mitochondrial number accompanied by a marked reduction in overall mitochondrial mass and reduced sarcomeric length, hallmarks of pathological mitochondrial fragmentation [53, 54]. This phenotype mirrors the mitochondrial fission observed in ischemic heart disease, where excessive fragmentation disrupts bioenergetic efficiency and promotes cardiomyocyte dysfunction [54, 55]. The presence of large cytoplasmic vacuoles and multivesicular structures further suggests impaired autophagic flux or stalled mitophagy, processes often linked to heart failure progression and compromised cellular quality control [56]. Together, these ultrastructural changes strengthen the concept that Letm1 overexpression induces maladaptive mitochondrial remodeling, which likely contributes to the deterioration of cardiomyocyte architecture and function under pathological stress.

### Role in autophagy, apoptosis, and cellular stress

Our findings establish that elevated Letm1 expression in cardiomyocytes initiates a cascade of stress responses that converge on dysregulated autophagy, impaired mitophagic clearance, and apoptosis, hallmarks of pathological remodeling. Proteomics data further substantiated these findings. Mitochondrial stress triggered by Letm1 overexpression led to the activation of upstream autophagy initiators, including AMPK and ULK1, as well as increased levels of phosphorylated ULK1 (Ser555). These molecular cues coincided with marked elevation of DRP1 and pDRP1-S616, indicating excessive mitochondrial fission, which, although protective under physiological conditions, can be detrimental when sustained and uncoupled from efficient mitophagy [54, 57].

Accumulation of LC3-II and p62 proteins, along with upregulated Parkin1 transcripts, suggests activation of autophagic and mitophagic machinery. However, the tandem GFP-RFP-LC3 assay revealed an accumulation of yellow puncta, particularly pronounced under Bafilomycin A1 treatment, indicating incomplete autophagic flux due to impaired lysosomal degradation. In support, LAMP2 protein levels were elevated, consistent with lysosomal burden rather than enhanced clearance. These disruptions point to a maladaptive autophagic response where mitophagy is initiated but not resolved, contributing to mitochondrial and cellular dysfunction [58].Functionally, this compromised autophagic clearance was associated with increased apoptosis, evidenced by elevated cleaved caspase-3 and 7. These findings are consistent with prior studies linking defective mitophagy and autophagic stalling to cardiomyocyte apoptosis and progression of heart failure [59, 60]. Letm1-overexpressing NRVCMs also exhibited elevated ROS levels, which likely act as upstream triggers for autophagy and cell death pathways, especially under hypoxic or ischemic stress.

Transcriptomic profiling provided further mechanistic insight into these stress responses. Key regulators of mitochondrial dynamics, cell cycle arrest, and stress adaptation were upregulated, reinforcing the pathological phenotype. Notably, Cdkn1, a cyclin-dependent kinase inhibitor associated with cardiomyocyte senescence and hypertrophy, was elevated, potentially contributing to reduced proliferation and increased vulnerability to stress [61]. Gdf15, a cytokine elevated in ischemic heart disease and heart failure, was significantly upregulated, highlighting the activation of inflammatory and metabolic stress pathways [62]. In parallel, Foxo4, a transcription factor known to regulate oxidative stress, autophagy, and apoptosis, was also induced. Foxo4 activation has been linked to maladaptive autophagy and mitochondrial turnover under nutrient deprivation and mitochondrial dysfunction [63, 64]suggesting its involvement in mediating Letm1-induced stress. Indeed, Foxo4 can enhance transcription of autophagy-related genes like p62 and LC3, which were elevated in our study, further supporting this hypothesis.

Together, these data outline a multi-layered stress response induced by Letm1 overexpression, initiated by mitochondrial dysfunction and sustained by dysregulated autophagy and apoptosis. The maladaptive increase in mitochondrial fragmentation, impaired autophagic flux, enhanced ROS generation, and activation of death pathways mirrors pathological processes observed in ischemia-reperfusion injury and heart failure [65]. These results underscore Letm1 as a potential modulator of cardiomyocyte viability, mitochondrial quality control, electrophysiological regulation and metabolic remodeling.

### Clinical implications and future directions

Interestingly, although both ischemic cardiomyopathy (ICM) and hypertrophic heart disease (HCM) involve mitochondrial dysfunction and cardiomyocyte stress-induced apoptosis, our findings reveal that Letm1 expression is selectively upregulated in ICM but remains unchanged in HCM. This divergence likely reflects fundamental differences in the etiology, metabolic context, and nature of mitochondrial stress between the two pathologies. Ischemic cardiomyopathy is characterized by acute energy deprivation, hypoxia, and mitochondrial depolarization, all of which are known to activate mitochondrial calcium overload, ROS production, and apoptotic cascades. Letm1, being a mitochondrial inner membrane protein involved in Ca^2+^ and K^+^ exchange, may be transcriptionally or post-translationally upregulated in response to these specific forms of mitochondrial destabilization as a maladaptive stress response to maintain ion homeostasis or compensate for membrane potential loss.

In contrast, hypertrophic remodeling involves more chronic, adaptive mitochondrial adaptations to support increased workload, often without the same degree of oxygen deprivation or mitochondrial collapse seen in ICM. The lack of Letm1 induction in HCM may reflect a preserved mitochondrial membrane potential and a less acute need for Letm1-mediated ion exchange, suggesting that Letm1 upregulation is more closely tied to ischemic stress and bioenergetic crisis rather than generalized hypertrophic remodeling. The pathological effects of Letm1 were further amplified under hypoxia, suggesting its role in exacerbating ischemic injury. This highlights the potential of targeting Letm1 as a therapeutic strategy to mitigate ischemic damage and preserve cardiac function. Future studies are needed to investigate the upstream regulators of Letm1 expression and its downstream signaling pathways to identify intervention points for modulating its activity. Moreover, in vivo models are needed to validate the translational relevance of Letm1-targeted therapies in improving cardiac outcomes post-ischemia.

In summary, this preliminary preclinical study provides novel insights into the multifaceted role of Letm1 in cardiac pathophysiology. By elucidating its impact on mitochondrial function, ion homeostasis, cardiomyocyte contractility, and cell survival, we advance the understanding of Letm1’s possible contribution to ischemic heart disease, laying the foundation for future therapeutic strategies.

### Limitations of the study

This study provides important mechanistic insights into the pathological consequences of elevated Letm1 expression in cardiomyocytes, particularly in relation to mitochondrial bioenergetics, calcium handling, autophagy, and cell viability. By employing neonatal rat cardiomyocytes, adult mouse cardiomyocytes, and human iPSC-derived cardiomyocytes, we have strengthened the translational relevance and developmental robustness of our findings. However, several limitations remain.

First, while our models encompass both immature and mature cardiomyocyte systems in vitro, they still lack the full physiological complexity of the in vivo cardiac environment, including mechanical loading, neurohumoral regulation, and systemic metabolic influences. Additionally, although adult mouse cardiomyocytes were included, their limited viability and transfection efficiency in culture may underrepresent certain functional or chronic outcomes of Letm1 overexpression. Furthermore, while our study identifies key molecular and functional consequences of Letm1 dysregulation, the long-term in vivo impact on cardiac structure and function, particularly in the context of disease models such as ischemia or heart failure, remains to be fully elucidated. Future studies using inducible and tissue-specific Letm1 overexpression or knockout mouse models, combined with in vivo functional assessments, will be essential to validate and extend these findings.

Nonetheless, our results provide compelling evidence that Letm1 plays a central role in modulating mitochondrial homeostasis and cardiac remodeling, forming a foundation for deeper exploration of its potential as a therapeutic target in cardiovascular disease.

## Supplementary Information

Supplementary Material 1.

Supplementary Material 2.

Supplementary Material 3.

## Data Availability

Data is provided within the manuscript or supplementary information files.

## Abbreviations

CVD: Cardiovascular diseases
Letm1: Leucine zipper EF hand containing protein 1
LC3: Microtubule-Associated Proteins 1 A/1B Light Chain 3 A
p62: Phosphotyrosine-Independent Ligand For The Lck SH2 Domain Of 62 KDa
Cpt1a: Carnitine Palmitoyltransferase 1 A
Glut1: Solute Carrier Family 2 Member 1, Glucose transporter type 1
PINK1: PTEN Induced Kinase 1
Foxo4: Forkhead Box O4
RYR2: Ryanodine receptor 2
SERCA2a: Sarco-Endoplasmic Calcium ATPase
qPCR: Quantitative real time polymerase chain reaction
OXPHOS: Oxidative Phosphorylation
APD50: Action potential duration at 50% repolarization
APD90: Action potential duration at 90% repolarization
ATP: Adenosine Triphosphate
WHS: Wolf Hirschhorn Syndrome
HEK293A: Human embryonic kidney cells
TMRM: Tetramethylrhodamine, methyl ester
TMRE: Tetramethylrhodamine, Ethyl Ester, Perchlorate
mΔΨm: mitochondrial membrane potential
ORAB: O-ring aortic banding
LAD: Left anterior descending artery ligation
HCM: Hypertrophic cardiomyopathy
HF: Heart failure
ICM: Ischemic cardiomyopathy
Nppa: Natriuretic peptide A
Nppb: Natriuretic peptide B
ETC: Electron transport chain
KEGG: Kyoto Encyclopedia of Genes and Genomes
GO: Gene ontology
ROS: Reactive oxygen species
MitoSOX: Mitochondrial superoxide indicator
HBSS: Hanks’ Balanced Salt Solution
AMCM: Adult mouse cardiomyocytes
iPSC: Induced pluripotent stem cells
ULK1: Unc-51-like autophagy activating kinase 1
LAMP2: Lysosome-associated membrane protein 2
AMPK: AMP-activated protein kinase
TEM: Transmission electron microscopy
