# Supplementary material for: Elevated levels of Letm1 drives mitochondrial dysfunction and cardiomyocyte stress-mediated apoptosis in cultured cardiomyocytes
Source: Cell Commun Signal. 2025 Aug 23;23:378. doi: 10.1186/s12964-025-02378-7 (PMC12374280; doi:10.1186/s12964-025-02378-7)
Supplement: Supplementary file 2 — Supplementary Material 2. [file 12964_2025_2378_MOESM2_ESM.docx]

Elevated levels of Letm1 drives mitochondrial dysfunction and cardiomyocyte stress-mediated apoptosis in cultured cardiomyocytes

Anushka Deshpande^1,2^, Leo Weirauch^1,2^, Tapan Kumar Baral^1,2^, Marco Steier^1,2^, Ankush Borlepawar^3^, Manju Kumari^1,2^, Lucia S Kilian^4,5^, Karsten Richter^6^, Elke Hammer^7,8^, Derk Frank^4,5^, Constanze Schmidt^1,2^, Norbert Frey^1,2^, Ashraf Y Rangrez^1,2,^*

^1^ Department of Cardiology, Angiology and Pneumology, Internal Medicine III, University Hospital Heidelberg, 69120 Heidelberg, Germany

^2^ DZHK (German Centre for Cardiovascular Research), partner site Heidelberg/Mannheim, 69120 Heidelberg, Germany

^3^ Medical school, Hamburg, Germany

^4^ Department of Cardiology and Internal Intensive Medicine, Internal Medicine III, University Hospital of Schleswig-Holstein, Kiel, Germany

^5^ DZHK (German Centre for Cardiovascular Research), partner site Kiel/Hamburg/Lübeck, 24105 Kiel, Germany

^6^ Core Facility Electron Microscopy, German Cancer Research Center (DKFZ), 69120 Heidelberg

^7^ Interfaculty Institute of Genetics and Functional Genomics, University Medicine Greifswald,, 17475 Greifswald, Germany

^8^ DZHK (German Centre for Cardiovascular Research), Partnersite Greifswald, 17475 Greifswald, Germany

* Corresponding author

[ashrafyusuf.rangrez@med.uni-heidelberg.de](mailto:ashrafyusuf.rangrez@med.uni-heidelberg.de);

Supplementary figure 1: A. Multitissue distribution pattern of Letm1 in mouse organs detected by quantitative real-time PCR (n=5). B. Co-Immunostaining of adult mouse cardiomyocytes with Mitotracker red and Letm1 in green, nuclei detected using DAPI. C. Im
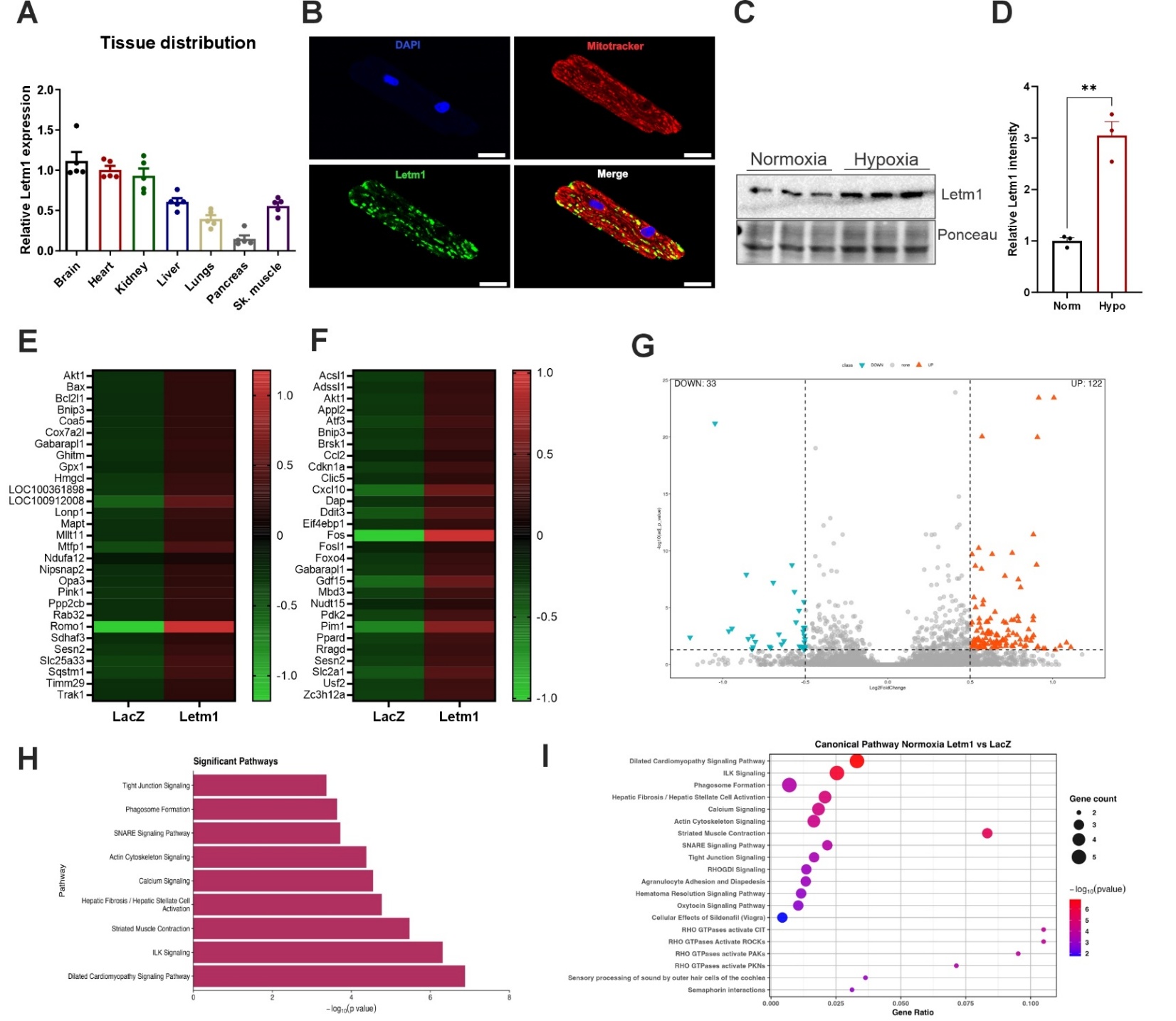
munoblot indicating Letm1 levels in NRVCMS under normoxia or hypoxia conditions. Its densitometry is shown in (D). E. Heat maps exhibiting upregulated genes involved in mitochondrial organization and (F) response to extracellular stimuli. G. Volcano plot from the proteomics analysis indicating differentially regulated genes upon Letm1. H. Bar plot of signinficanlty regulated pathways obtained from proteomics analysis and dot plot depicted in (I). n=3 for every experimental condition. Statistical significance is calculated by non-parametric Students’ t-test. **, p<0.01.


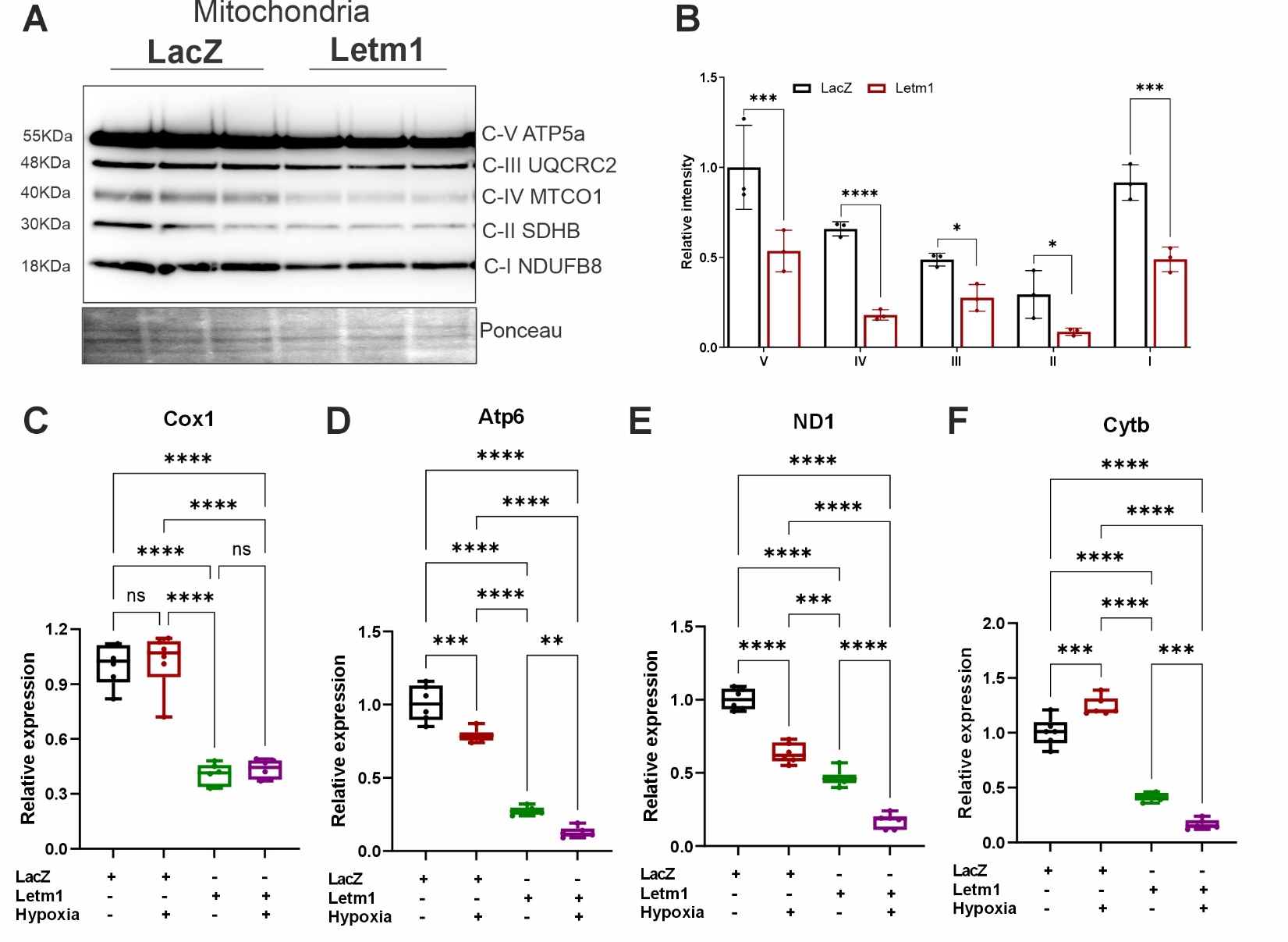


Supplementary figure 2: A. Immunoblotting of OXPHOS complexes in isolated mitochondrial samples from NRVCMs overexpressing Letm1 as compared to LacZ and its densitometric analysis in B. C-F. Transcript level analysis of genes involved in mitochondrial electron transport chain upon Letm1 in presence hypoxia as compared to normoxia condition. n=3 for every experimental condition. Statistical significance is calculated by non-parametric Students’ t-test or 2-way ANOVA. *, p<0.05 **, p<0.01, ***, p<0.001, ****, p<0.0001.


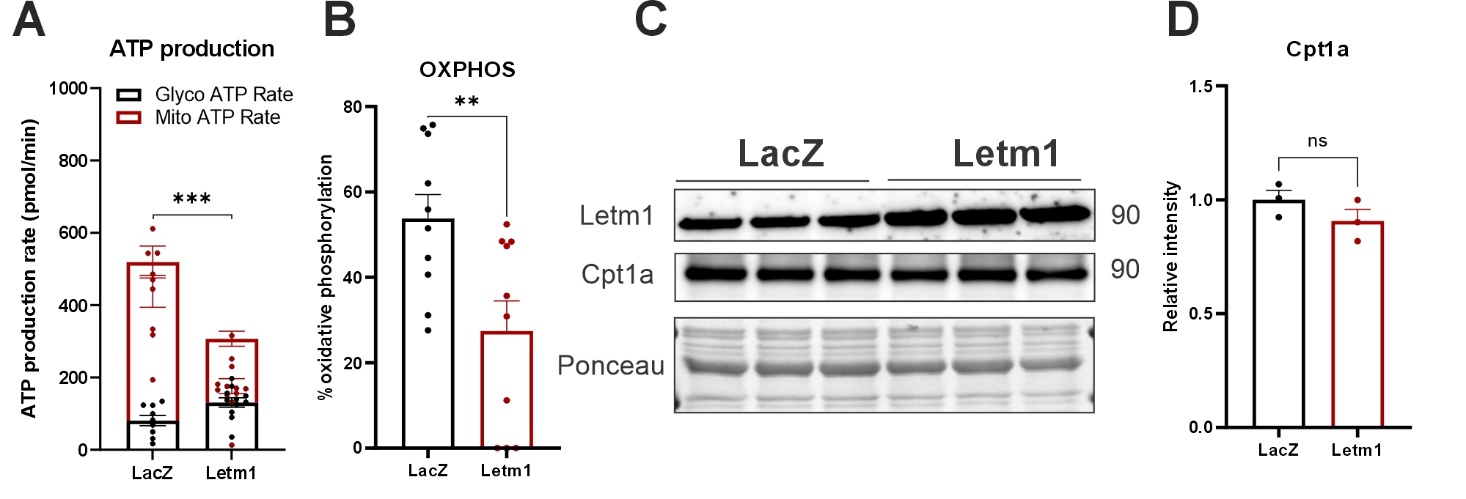


Supplementary figure 3: A. ATP production graph from adult mouse cardiomyocytes plotted using measurements from ATP rate assay to calculate ATP obtained from mitochondrial vs glycolysis pathway. B. reduced % of oxidative phosphorylation calculated from the ATP rate assay upon Letm1 overexpression. C. Immunoblot and its densitometry (D) depicting Cpt1a levels in AMCMs expressing Letm1 compared to LacZ control.


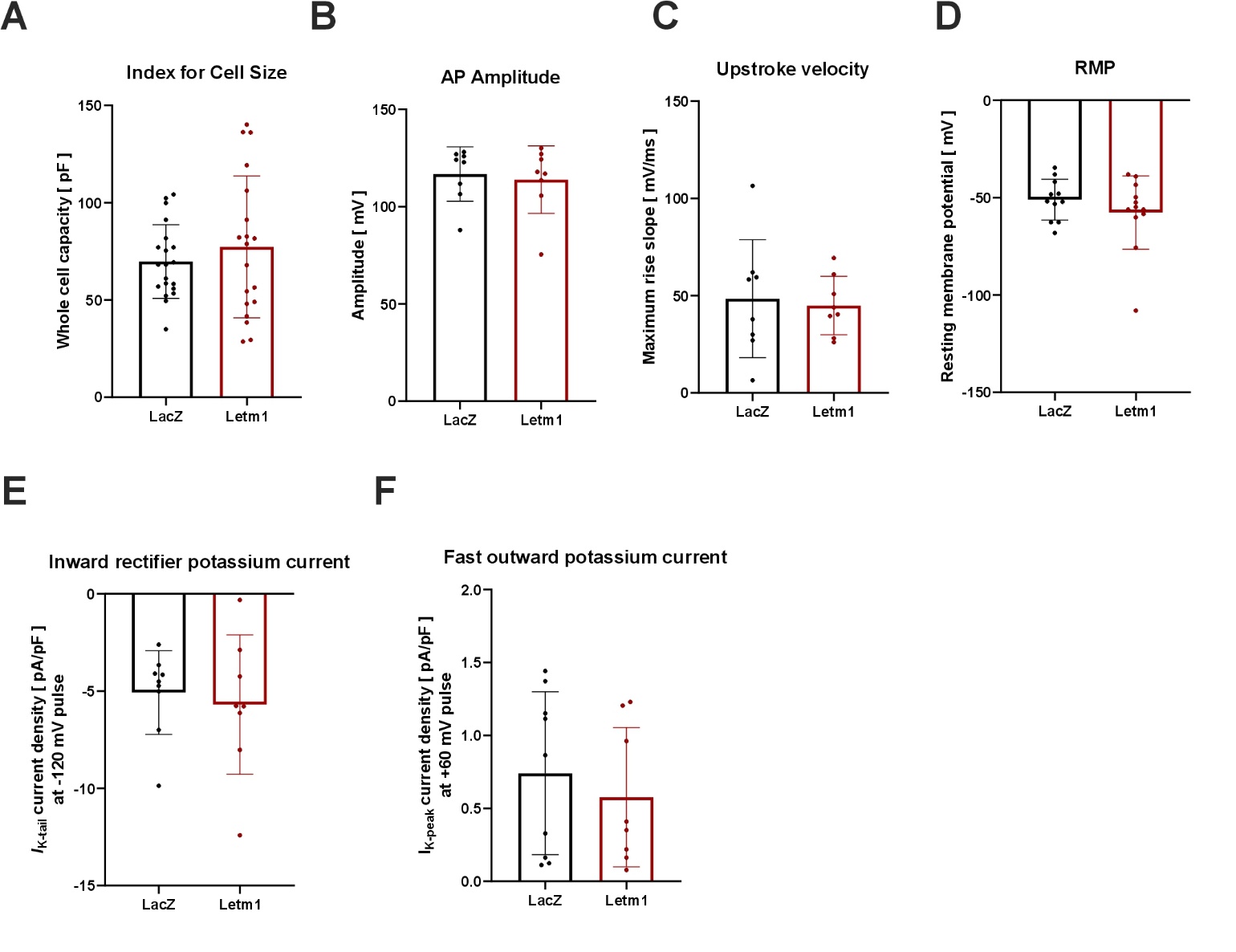


Supplementary figure 4: A-D. Bar graphs indicating cell size, action potential amplitude, upstroke velocity and resting membrane potential obtained from patch clamp experimental analysis in NRVCMs expressing either Letm1 or LacZ. E-F. Potassium current measurements of inward and outward current upon Letm1 expression as compared to LacZ control. Statistical significance is calculated by non-parametric Students’ t-test. None of the parameters are statistically significant.


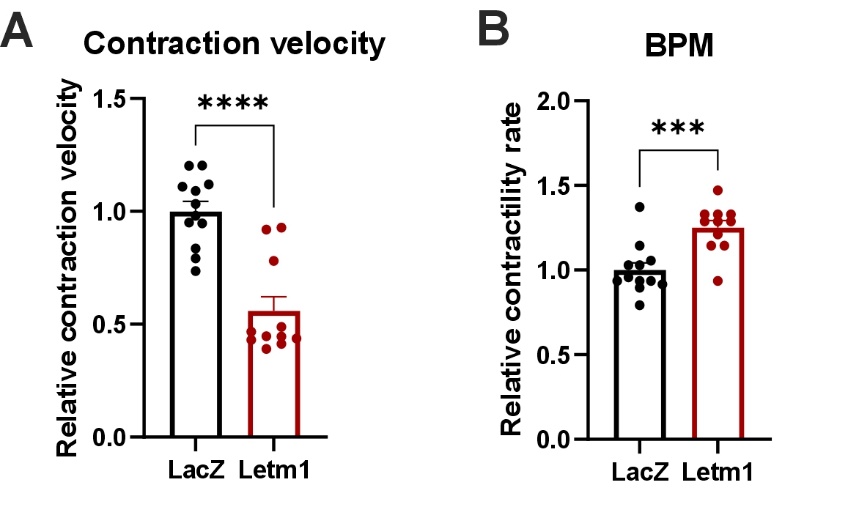


Supplementary figure 5: Contraction velocity (A) and cardiomyocyte beating rate (B) determined from the contractility assay. Statistical significance is calculated by non-parametric Students’ t-test. ***, p<0.001, ****, p<0.0001.


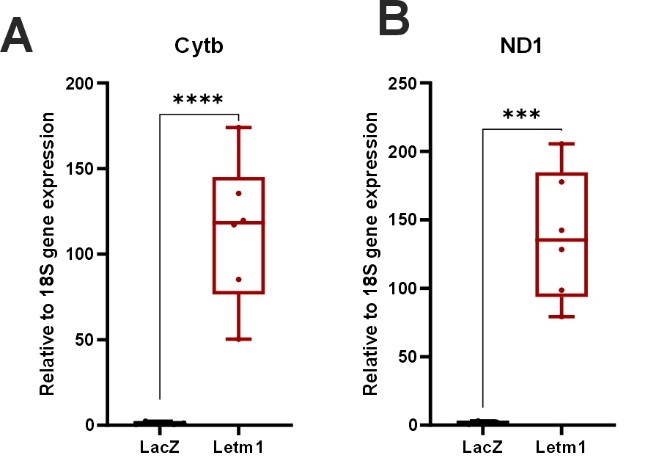


Supplementary Figure 6: A-B. Increased mitochondrial copy number in NRVCMs overexpression Letm1 normalized to nuclear 18S gene as compared to mitochondrial Cytb and ND1 gene. Statistical significance is calculated by non-parametric Students’ t-test. ***, p<0.001, ****, p<0.0001.


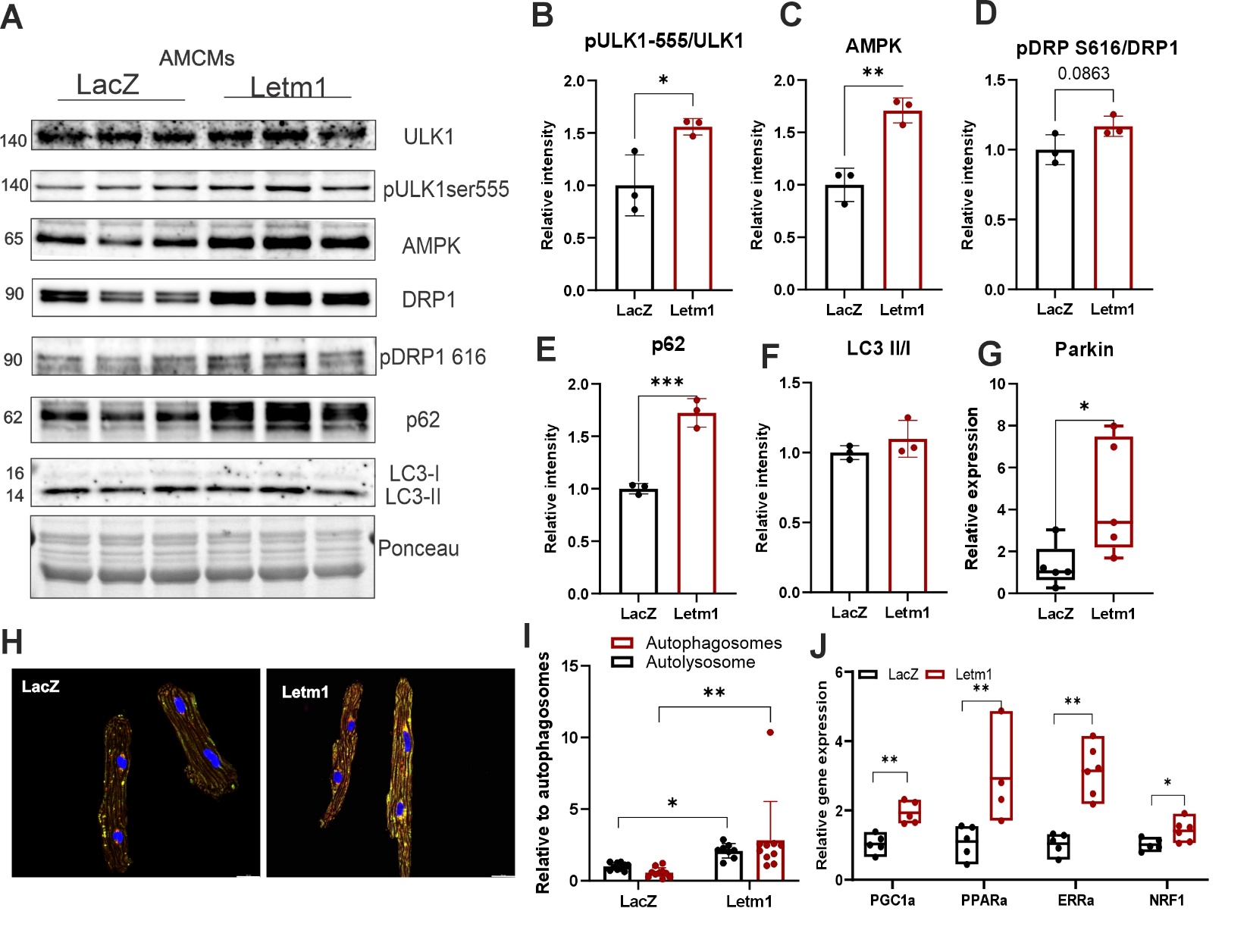


Supplementary figure 7: A. Immunoblots indicating different markers for AMPK-ULK1 signaling pathway, mitochondrial fission and autophagy in Letm1 overexpressing AMCMs as compared to LacZ control condition along with their respective densitometric analysis in (B-F). G. Increased transcript level of Parkin upon Letm1 elevation. H. Representative images of Tandem LC3-GFP-RFP imaging in cells with increased Letm1 and its analysis in I. J. Transcript levels of transcription factors responsible for mitochondrial biogenesis upon increased Letm1 in AMCMs. n=3. Statistical significance is calculated by non-parametric Students’ t-test or 2 way ANOVA. *, p<0.05; **, p<0.01; ***, p<0.001; ****, p<0.0001; ns, non-significant.


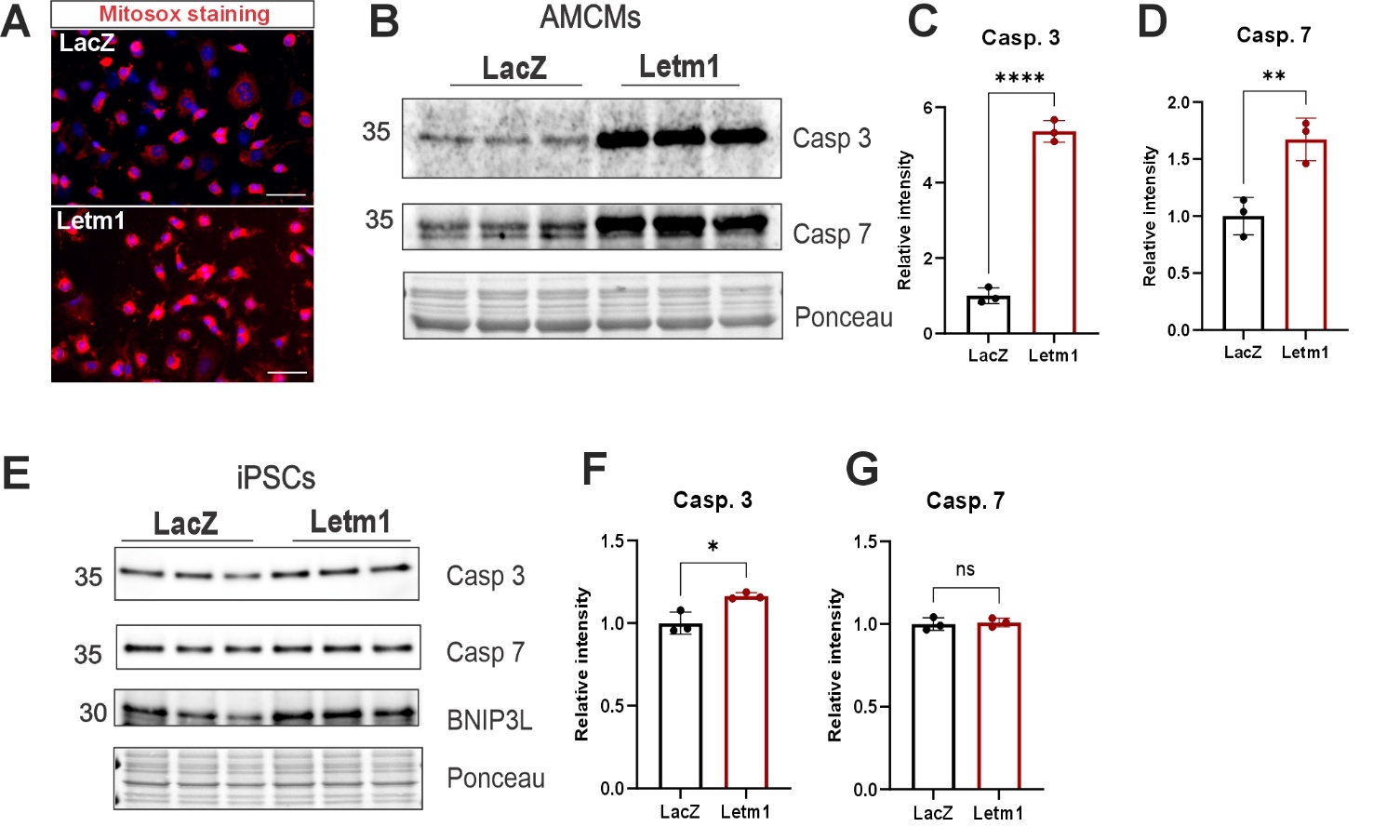


Supplementary figure 8: A. Representative immunofluorescence images of cardimyocytes expressing either Letm1 or LacZ control, stained with Mitosox dye for determination of reactive oxygen species (Scalebar 100 µm). Immunoblots presenting expression levels of Casp 3 and 7 (B), and respective densitometry analysis shown in bar graphs (C-D) in AMCMs. Immunoblots presenting expression levels of Casp 3 and 7 and increased BNIP3L (E), and respective densitometry analysis shown in bar graphs (F,G) in iPS dereived cardiomycoytes. Statistical significance is calculated by non-parametric Students’ t-test. *, p<0.05, **, p<0.01, ***, p<0.001.
