# Supplementary figures and images for: Elevated levels of Letm1 drives mitochondrial dysfunction and cardiomyocyte stress-mediated apoptosis in cultured cardiomyocytes
Source: Cell Commun Signal. 2025 Aug 23;23:378. doi: 10.1186/s12964-025-02378-7 (PMC12374280; doi:10.1186/s12964-025-02378-7)

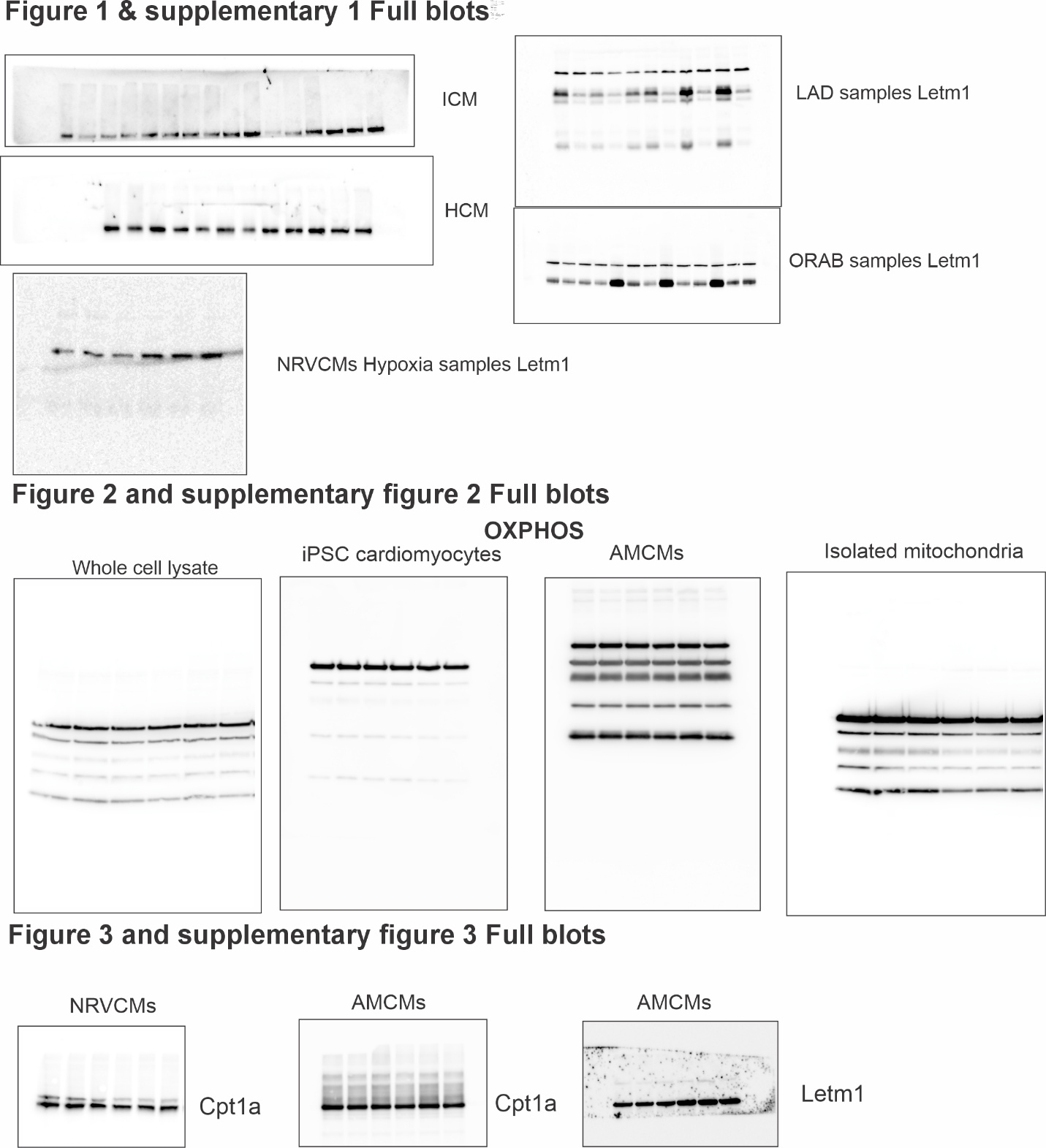


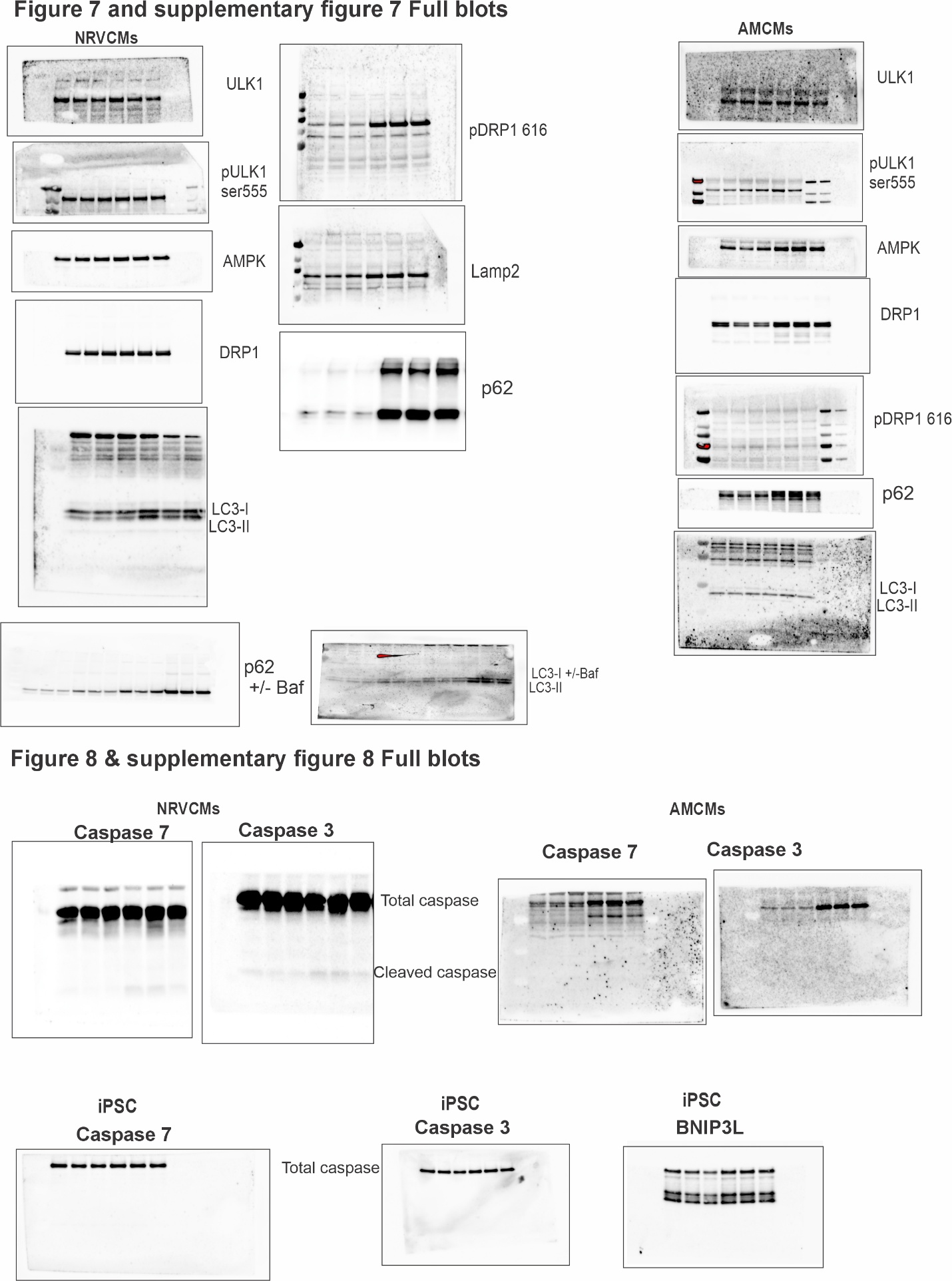

Supplement: Supplementary file 3 — Supplementary Material 3. [file 12964_2025_2378_MOESM3_ESM.docx]
